# Identification and Genome Analysis of an Arsenic-Metabolizing Strain of *Citrobacter youngae* IITK SM2 in Middle Indo-Gangetic Plain Groundwater

**DOI:** 10.1155/2022/6384742

**Published:** 2022-03-10

**Authors:** Akshat Verma, Prem Anand Murugan, Hariharan Vedi Chinnasamy, Abhas Singh, Saravanan Matheshwaran

**Affiliations:** ^1^Department of Civil Engineering, Indian Institute of Technology Kanpur, India; ^2^Environmental Geochemistry Laboratory, Centre for Environmental Science and Engineering, Indian Institute of Technology Kanpur, India; ^3^Department of Biological Sciences and Bioengineering, Indian Institute of Technology Kanpur, India; ^4^Environmental Microbiology Laboratory, Center for Environmental Science and Engineering, Indian Institute of Technology Kanpur, India; ^5^Mehta Family Center for Engineering and Medicine, Indian Institute of Technology Kanpur, India

## Abstract

Whole-genome sequencing (WGS) data of a bacterial strain IITK SM2 isolated from an aquifer located in the middle Indo-Gangetic plain is reported here, along with its physiological, morphological, biochemical, and redox-transformation characteristics in the presence of dissolved arsenic (As). The aquifer exhibits oxidizing conditions relative to As speciation. Analyses based on *16S rRNA* and *recN* sequences indicate that IITK SM2 was clustered with *C. youngae* NCTC 13708^T^ and *C. pasteuri* NCTC UMH17^T^. However, WGS analyses using the digital DNA-DNA hybridization and Rapid Annotations using Subsystems Technology suggest that IITK SM2 belongs to a strain of *C. youngae*. This strain can effectively reduce As(V) to As(III) but cannot oxidize As(III) to As(V). It exhibited high resistance to As(V) [32,000 mg L^−1^] and As(III) [1,100 mg L^−1^], along with certain other heavy metals typically found in contaminated groundwater. WGS analysis also indicates the presence of As-metabolizing genes such as *ars*C, *ars*B, *ars*A, *ars*D, *ars*R, and *ars*H in this strain. Although these genes have been identified in several As(V)-reducers, the clustering of these genes in the forms of *ars*ACBADR, *ars*CBRH, and an independent *ars*C gene has not been observed in any other *Citrobacter* species or other selected As(V)-reducing strains of *Enterobacteriaceae* family. Moreover, there were differences in the number of genes corresponding to membrane transporters, virulence and defense, motility, protein metabolism, phages, prophages, and transposable elements in IITK SM2 when compared to other strains. This genomic dataset will facilitate subsequent molecular and biochemical analyses of strain IITK SM2 to identify the reasons for high arsenic resistance in *Citrobacter youngae* and understand its role in As mobilization in middle Indo-Gangetic plain aquifers.

## 1. Introduction

The *Citrobacter* genus, which belongs to the *Enterobacteriaceae* family, was first described in 1932 [1]. Till now, 18 species of *Citrobacter* have been identified [2, 3] from varied sources like soil, water, sewage, feces, and intestinal gut of animals and humans [4, 5]. Members of *Citrobacter* are enteric Gram-negative and rod-shaped coliform bacteria with 1.0 × 2.0–6.0 *μ*m in size [6]. Some strains of *Citrobacter* are opportunistic pathogens and can cause infections in immunocompromised patients [7]. It is often reported that *Citrobacter* species are the cause of meningitis in infants [8]. Among the different species of *Citrobacter*, *C. youngae* causes inflammation in the peritoneum, the membrane that covers the inner wall of the abdomen [9]. Furthermore, different strains of *Citrobacter* are known to be resistant to several heavy metals, including arsenic (As; [10, 11]).

Arsenic (As) is a geogenic metalloid contaminant that has affected the health of animals and humans [12–15]. Sustained consumption of As-polluted water [>10 *μ*g/L, WHO permissible limit [16]] can cause acute to chronic health problems in humans [13, 15]. Depending on its electronic configuration, As can exhibit multiple oxidation states: +V [arsenates; As(V)], +III [arsenites; As(III)], 0, and –III [15, 17]. In nature, +V and +III are the predominant oxidation states of As [14]. These arsenic species can exist in inorganic (^i^As^V^/^III^) and methylated (^m^As^V^/^III^) forms [18]. In mammalian systems, As(III) is identified as more toxic than As(V) [19]. Monomethylated arsenous acid [MMA(III)] is considered to be the most toxic of all forms of As(V) and As(III) [20, 21]. However, MMA(III) and other ^m^As forms, such as monomethylated arsenic acid [MMA(V)] and dimethylated As species, DMA(III), and DMA(V), are generally not detected in food or water. At times they are found in urine or its by-products [22, 23].

The ^i^As^V^/^III^ forms are the major forms of As present in groundwater [15]. In aquifers, As redox transformations can be affected by site-specific conditions such as the prevalent pH, redox potential (E_H_), co-ions, and the presence of labile organic matter [12, 14]. Although the source of arsenic pollution in groundwater is mainly inorganic [24–26], involvement of the indigenous microbial population in As-redox transformations and mobilization cannot be ignored if abundant labile organic matter was present [18, 27]. These microbes can metabolize arsenic due to the presence of As-metabolizing genes in their system [18, 19]. These As-metabolizing organisms are classified as (a) As(V) reducers [28, 29] and/or (b) As(III) oxidizers [30, 31]. The presence of these organisms in groundwater could indicate microbially mediated arsenic transformation and resultant speciation [27]. Specifically, As(V) reducers have the potential to control As concentrations in the groundwater due to their capacity to bioreduce host minerals of As(V) [32]. The bacterial genera known for their efficient reduction of As(V) to As(III) are S*ulfurospirillum*, *Bacillus*, *Wolinella*, *Clostridium*, *Staphylococcus*, *Desulfomicrobium*, and *Citrobacter* [33–35].

In this study, a strain of *Citrobacter youngae* IITK SM2 (hereafter referred to as strain “IITK SM2”) was isolated from middle Indo-Gangetic plain (IGP) groundwater in India, at conditions that were oxidizing with respect to arsenic speciation. The objectives of the study were as follows: (i) identification of major genes, including As-resistant genes, present in this strain through whole-genome sequencing (WGS); (i) determination of physiological, morphological, biochemical, and redox-transformation characteristics of this isolate in the presence of dissolved arsenic; and (iii) identification of features in the arsenic-operon system contained in this strain relative to other organisms. To the best of our knowledge, this is the first strain of *Citrobacter youngae* which shows effective As(V) reduction to As(III). This information will be helpful in the identification of the As(V) metabolizing enzymes and other proteins involved in the influx and efflux of As relative to this bacteria.

## 2. Materials and Methods

### 2.1. Study Site, Sample Collection, and Analysis

Groundwater was sampled from a previously identified As-polluted aquifer in Baikunthpur, Uttar Pradesh, India (26°33′47.3^″^N and 80°15′18.5^″^E), situated in the Indo-Gangetic plains [[36]; Figure S1 of the supporting information]. Two different groundwaters, As-polluted and As-unpolluted, were sampled from this site (Figure S1). For each sample, pH, temperature (°C), conductivity (*μ*S cm^−1^), and redox potential (E_H_; V) were measured at the site using a portable multiparameter meter (Thermo Orion Star A329) and suitable electrodes. Three sets of water samples were collected from the polluted aquifer. Two sets were filtered using 0.2 *μ*m nylon syringe filters (Cole-Parmer). One of these filtered samples was immediately acidified using 1% (*v*/*v*) trace-metal grade HNO_3_ for the determination of dissolved total arsenic (As_T_) and other elements using inductively coupled plasma mass spectrometry (ICP-MS). The other set of filtered samples was left unacidified and was collected without headspace in 15 mL centrifuge tubes. This filtered-unacidified sample set was utilized for dissolved carbon analysis using total organic carbon (TOC) analyzer, and for the measurement of dissolved inorganic As(V) and As(III) using ion chromatography coupled with ICP-MS (IC-ICP-MS). Due to the reported inaccuracies in measured redox potential values using electrode [37–41], E_H_ was also estimated using measured As(V) and As(III) concentrations using the Nernst equation for As-polluted groundwater [42] (Section S1 of the Supporting Information).

For bacterial culturing, unfiltered groundwater samples were collected in sterilized tubes without headspace and were sealed with Parafilm. All these samples were placed in ice gel packs and transported within 1 h to the laboratory. Subsequently, the unfiltered samples were transferred to predefined As-amended agar plates inside a laminar hood.

### 2.2. Chemicals

All solutions and buffers were prepared in ultrapure water (Milli-Q, resistivity > 18.2 M*Ω* − cm) and were either filtered using 0.2 *μ*m nylon syringe filters or autoclaved at 121°C for 20 min before use. For preparing 100 mg mL^−1^ stock solutions of As(V) and As(III), Na_2_HAsO_4_·7H_2_O and NaAsO_2_, respectively, were used. For qualitative As redox transformation test, a stock solution of 1 M AgNO_3_ was prepared prior to use and was stored in dark at 4°C. Chemicals used in this study, and their manufacturers and purities, are detailed in Table S1 of the Supporting Information.

### 2.3. Analytical Techniques

Elemental concentrations, including total dissolved arsenic (As_T_), were measured using inductively coupled plasma mass spectrometry (ICP-MS; Thermo iCAP-Qc), with germanium as an internal standard for As measurement. All standards and samples were analyzed in a 1% HNO_3_ matrix. Dissolved As(V) and As(III) concentrations were measured using ion chromatography coupled with ICP-MS (IC-ICP-MS; Thermo Scientific iCAP Q with the Thermo Scientific Dionex ICS-5000 IC). Analytes were eluted in 100 mM (NH_4_)_2_CO_3_ over IonPac AS7 analytical column. Dissolved total carbon (TC) and inorganic carbon (DIC) concentrations were measured with total organic carbon analyzer (TOC-L; Shimadzu TNM-L ROHS), and dissolved TOC was estimated from TC and DIC (TOC = TC–DIC). The optical density of cultures was monitored by the measurement of absorbance at 600 nm (OD_600_; Biospectrometer; Eppendorf). The method detection limits of various techniques are listed in Table S2 of the Supporting Information.

Morphology of the bacterial strain was determined microscopically. The Gram-staining test followed by optical microscopy was performed at 100x magnification (Quasmo; Ecostar-plus). For higher magnification and better resolution, tungsten scanning electron microscopy with associated energy dispersive X-ray spectroscopy (W-SEM-EDX; JEOL JSM 6010 LA) was used. Before SEM-EDX analysis, samples were gold-coated at 7-10 nm.

### 2.4. Isolation of Arsenic-Resistant Bacteria

To isolate As-resistant bacteria, 100 *μ*L of As-polluted groundwater was added to each of the As-amended Lysogeny agar plates (0-400 mg L^−1^) and spread using sterilized glass beads in a laminar flow hood. These plates were incubated at 37°C for 24 h, and sixteen distinct colonies were isolated upon visual identification. However, only two isolates grew in 400 mg L^−1^ each of As(III)- and As(V)-containing Lysogeny agar plates. Of these isolates, the strain (IITK SM2) which showed more efficient growth in the presence of arsenic was selected for WGS. Strain IITK SM2 was inoculated in Lysogeny broth (LB) and diluted several times by streaking the culture on variables As-supplemented (0-10,000 mg L^−1^) Lysogeny agar plates until single colonies were obtained. For further analyses, these cultures were preserved in 12–15% glycerol solution at -80°C.

### 2.5. Morphological, Physiological, and Biochemical Characterization of Strain IITK SM2

Experiments were performed with strain IITK SM2 to determine its bacterial group, optimum growth conditions, and response of bacterial growth to specific biochemical tests. Experimental details of these tests are provided in Section S2 of the Supporting Information.

#### 2.5.1. Effect of Arsenic on the Growth: Kinetics and Morphology

The growth profile of IITK SM2 was studied in the absence and presence of arsenic [As(V) or As(III)]. Before starting this experiment, it was confirmed that dissolved As was not present in the background media. Initially, a single colony of this isolate from Lysogeny agar plate was inoculated at 30°C (optimum temperature) and 120 rpm in arsenic-free minimum salt media (MSM; Table S3 of the Supporting Information), supplemented with 10 mM glucose as the only C source. Optical absorbance at 600 nm (OD_600_) of this culture was regularly recorded (BioSpectrometer® basic; Eppendorf) until an OD_600_ of ~0.1 was reached. Thereafter, ~1% of the bacterial suspension was individually transferred to set-up different cultures which contained 0 mM, 1.33 mM, 3.33 mM, 6.67 mM, 10 mM, 13.33 mM, and 20 mM of either As(V) or As(III) in 10 mM glucose-supplemented MSM. These cultures were incubated at 30°C and 120 rpm. After 8 h of transfer, the OD_600_ was measured for all the cultures. This experiment was performed three times in triplicate. Among all the concentrations investigated, the maximum biomass was obtained at 10 mM of As(V) dosage (Figure S2 of the Supporting Information). Consequently, 10 mM of As was chosen as the optimum dosage for comparative growth profile study in the presence of either 10 mM As(V) or 10 mM As(III) along with the As-free control. Doubling times for all these three cultures [10 mM As(V), 10 mM As(III), and 0 mM As] were calculated from respective growth curves by considering two log-unit increase in bacterial population. For these three cultures, morphological characterization of bacterial cells was performed using SEM. Details of sample preparation for SEM analysis are discussed in the section S3 of the Supporting Information.

#### 2.5.2. Resistance to As and Other Heavy Metals Typically Present in Contaminated Groundwater

Minimum inhibitory concentration (MIC) tests were performed to evaluate the resistance of strain IITK SM2 to As(III) and As(V), along with certain heavy metals found in contaminated groundwaters like Fe(III), Cr(VI), Mn(II), Ba(II), and Zn(II).The MIC was defined as the lowest metal concentration at which no bacterial growth was observed [43]. The detailed procedure used for determining MIC is discussed in the section S4 of the Supporting Information.

### 2.6. Redox Transformation of Arsenic

The capability of strain IITK SM2 to transform As(V) to As(III) or vice versa was qualitatively estimated by the formation of colored precipitates upon addition of silver nitrate, per a slightly modified procedure from what is detailed in a previous study [44]. This procedure is discussed briefly in Section S5 of the Supporting Information. Again, MSM supplemented with 10 mM glucose was used as the culturing media without any background As(V) and As(III). Arsenic was either added as As(V) or As(III) to obtain final concentrations as 0, 50, 100, and 500 mg L^−1^ of each form of arsenic. Also, systems without bacterial cultures were initiated as controls with the same set of As concentrations (0-500 mg L^−1^). For confirmation of dissolved arsenic concentrations, As_T_ was measured using ICP-MS, and As(V) and As(III) were measured using IC-ICP-MS before and after the addition of strain IITK SM2.

### 2.7. Molecular Characterization

To identify the genus and species of strain IITK SM2, *16*S*rRNA* and whole-genome sequencing were performed. The methods used for the isolation of genomic DNA and for 16S rRNA sequencing are detailed in Section S6 of the Supporting Information.

#### 2.7.1. Whole-Genome Sequencing (WGS)

Libraries were constructed in alignment with microbial WGS with the recommendations of Nextera™ DNA flex library preparation kit from Illumina Inc. To determine the mean fragment size, libraries were loaded and analyzed on a high-sensitivity D1000 ScreenTape. The Illumina libraries were diluted to 4 nM pooled, spiked with 5% PhiX, premade Illumina library, and loaded onto a Miseq v2 kit. The sequencing was performed for 2 x 150 cycles. The raw data obtained from Illumina MiSeq were recorded as FASTQ files. The adapter-free reads were obtained using an adapter trimming plugin. The quality check of the reads was done using FastQC v0.11.3 [45]. High-quality reads were obtained using Trimmomatic v0.39 [46]. *De novo* assembly and scaffolding were done using SPAdes v3.14.1 [47], where filtered reads were assembled without biasing the assembly to any known genome. The quality check was performed using QUAST v5.0.2 [48]. For assembly of the genome, only contigs with more than 500 bp were considered. The g-DNA sequences were assembled into 30 different contigs. The assembly was annotated using National Center for Biotechnology Information, Prokaryotic Genome Annotation Pipeline [[49]; NCBI-PGAP v2020-09-24 build4894)]. The g-DNA of the strain was mapped to reference organisms—*Citrobacter freundii* FDAARGOS 549 (GenBank accession number NZ_CP033744.1) and *Citrobacter youngae* NCTC 13708 (GenBank accession number NZ_UFWE01000006.1) using Bowtie 2 [50].

#### 2.7.2. Sequence Comparison of IITK SM2 with Different Strains

To identify whether the isolate belongs to a new species or not, type (strain) genome server (TYGS) using formula 2 was applied for the computation of digital DNA-DNA hybridization (dDDH) value [51]. Furthermore, subsystem features in strain IITK SM2 were also compared with features of different *Citrobacter* species using rapid annotations with subsystem technology [RAST, [52]. The different types of strains used for these analyses are detailed in Table S4 of the Supporting Information.

Based on the coding sequences (CDs), orthologous gene clusters were determined by comparing the genome of isolate IITK SM2 with genomes of closely related strains. These strains were chosen based on dDDH values. For this clustering, the OrthoVenn web server was used [53]. Default parameters such as e-value cut-off of 10^−5^and inflation value of 1.5 were used to compare protein similarity and to generate orthologous clusters, respectively.

Through the WGS, arsenic-resistant genes were also identified in the g-DNA of this strain. The arrangement of these genes, or *ars* operon in IITK SM2, was compared with genetic arrangement of different species of *Citrobacter* and selected strains of *Enterobacteriaceae* (Table S4 of the Supporting Information).

#### 2.7.3. Phylogenetic Analysis

The evolutionary relationship of strain IITK SM2 was determined by the comparison of its 16S rRNA sequence with sequences of closely related bacterial species. Furthermore, to accurately differentiate among different species, the highly conserved *recN* gene sequence was used. For these analyses, sequences of related bacterial strains were downloaded from the NCBI database using basic local alignment search tool [BLAST, [54]]. Multiple alignments of protein sequences of As(V)-reductase gene *arsC* identified in strain IITK SM2 were performed using CLUSTAL_W [55]. Also, to understand the evolutionary history of these genes, *ars*C sequences of our isolate were compared with sequences of bacterial species from different genera than of strain IITK SM2. Accession numbers of different strains used for these analyses are detailed in Table S4 of the Supporting Information.

The bootstrap method was used for testing the phylogeny with 1000 replicates [56]. Phylogenetic trees of different sequences mentioned above were prepared using the neighbor-joining [NJ] method [57]. For computing the evolutionary distances, the p-distance method was used [58] and the units were reported in base differences per site. MEGA X was used for conducting these evolutionary analyses [59].

## 3. Results and Discussion

### 3.1. Geochemistry of As-Polluted Aquifer

The As-polluted groundwater sampled from the study site exhibited oxidizing conditions with respect to arsenic speciation as suggested by the measured dissolved arsenic, co-solutes, and calculated redox potential (*E*_*H*_^*m*^~112 mV). On an average, ~70 *μ*g L^−1^ of total dissolved arsenic (As_T_) was recorded, of which the dissolved inorganic As(V) and dissolved inorganic As(III) concentrations were~43 *μ*g L^−1^ and~22 *μ*g L^−1^, respectively. Methylated forms of arsenic were not measured in groundwater because it is well documented in the literature that simple methylated forms such as MMA(III), DMA(V), MMA(V), and DMA(V) are usually not detected in food or water [15, 22, 23, 60, 61] but are detected in urine or its by-products [62–64]. Furthermore, the sum of these dissolved inorganic As(V) and As(III) was within the 10% of As_T_, which also suggests that methylated forms of arsenic, if present, were in negligible quantity in the groundwater of our interest (Table 1). Higher concentration of As(V) as compared to As(III) supports the prevalence of oxidising conditions with respect to arsenic speciation. However, the presence of significant concentration of the reduced form of arsenic [~22 *μ*g L^−1^ as As(III)] in such oxidizing aquifers indicated a potential role of As(V)-reducing organisms. Furthermore, high average DIC (~1296 mg L^−1^) and TOC (~40 mg L^−1^) concentrations as compared to unpolluted aquifer (DIC~186 mg L^−1^ and TOC~11 mg L^−1^) suggest the potential role of microbial activity in this groundwater. Other water quality parameters are detailed in Table 1.

One of the most widely accepted mechanism of arsenic mobilization in groundwater is reductive dissolution of iron (oxy)hydroxide. [FeOOH_(s)_] [24, 25, 65–73]. However, this mechanism is more prevalent under reducing conditions [66]. For the oxidizing groundwater from where the strain IITK SM2 was isolated as in this study, As(V) might still be released from reductive dissolution of FeOOH but the presence of As(III) in such aquifers hints towards the microbially mediated As(V)-reduction to As(III). However, a detailed and careful study would be required for identifying the role and resultant mechanisms of such microbes in arsenic speciation in oxidizing aquifers. This would require systematic investigation and comparison of indigenous microbial population from both As-free and As-polluted groundwaters.

### 3.2. Characterization of Bacterial Strain IITK SM2

#### 3.2.1. Classification of the Isolate

The isolate was rod-shaped Gram-negative, catalase-positive, and motile bacteria, which exhibited a negative starch hydrolysis test. Strain IITK SM2 can grow over pH 4–10, with an optimum pH of 7.25. Furthermore, the isolate grew over 15–45°C, with an optimum growth at 30°C at pH 7.25 (Figure S3 of the Supporting Information). The strain could tolerate NaCl up to 6% (*w*/*v*) and indicated an optimal growth at 1.5% (*w*/*v*) of the salt. After incubation for 2 d in an anaerobic chamber, colonies grown in Lysogeny agar plates were circular, opaque, and yellow, which suggested that the isolate was a facultative anaerobe. Furthermore, strain IITK SM2 showed resistance to ampicillin (100 *μ*g L^−1^) and hygromycin (50 *μ*g L^−1^), but growth was not observed in the presence of kanamycin (50 *μ*g L^−1^), or chloramphenicol (25 *μ*g L^−1^), or ciprofloxacin (20 *μ*g L^−1^), or gentamycin (10 *μ*g L^−1^), or streptomycin (50 *μ*g L^−1^).

**Figure 1:**
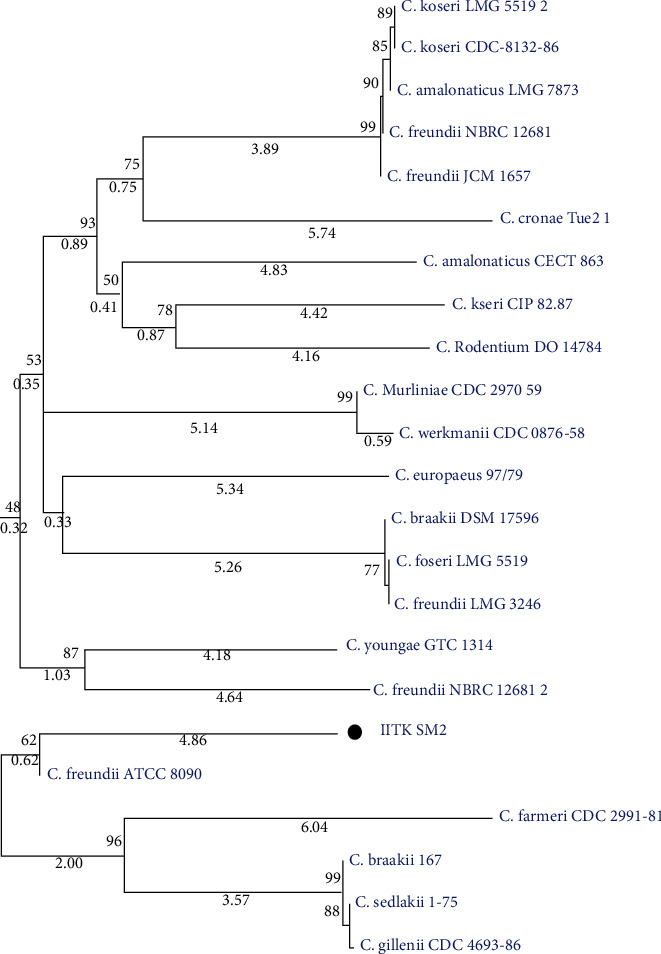
Phylogenetic position of strain IITK SM2 relative to other strains of genus *Citrobacter* based on *16S rRNA* sequences. The neighbor-joining method was used for tree construction [57]. Percentage bootstrap values corresponding to 1000 replicates are shown next to the branches in “bold”. Branch lengths are shown in “narrow italics” below each branch. The tree was drawn to scale.

The strain IITK SM2 belongs to genus *Citrobacter*, as determined by *16S rRNA* sequencing, and is closely related to *Citrobacter murliniae* CDC 2970-59^T^ (98.7%), *Citrobacter freundii* ATCC 8090^T^ (98.3%), *Citrobacter werkmanii* CDC 0876-58^T^ (98.2%), and *Citrobacter youngae* GTC 1314^T^ (97.8%). Furthermore, a distance-based phylogenetic tree construction revealed that the strain was clustered with *Citrobacter freundii* ATCC 8090 (Figure 1).

#### 3.2.2. Resistance to Heavy Metals

Among the metal species considered in this study, IITK SM2 showed resistance to As(V), As(III), Fe(III), Cr(VI), Mn(II), Ba(II), and Zn(II) up to certain levels (Table 2). Of these species, the maximum resistance was observed for As. The minimum inhibitory concentrations (MIC) of As(V) and As(III) were 32,000 mg L^−1^ (427 mM) and 1100 mg L^−1^ (~14.7 mM), respectively. To the best of our knowledge, such high MICs of As(III) and As(V) have not been reported for any other *Citrobacter* species till date. A comparison of MIC of Gram-negative rod-shaped arsenate reducers is made in Table S5 of the Supporting Information. Furthermore, the MIC for Fe(III), Mn(II), Cr(VI), Ba(II), and Zn(II) were estimated to be 17.9 mM, 91.0 mM, 0.2 mM, 14.7 mM, and 3.8 mM, respectively (Table 2).

#### 3.2.3. Impact of Arsenic on Growth and Morphology

**Figure 2:**
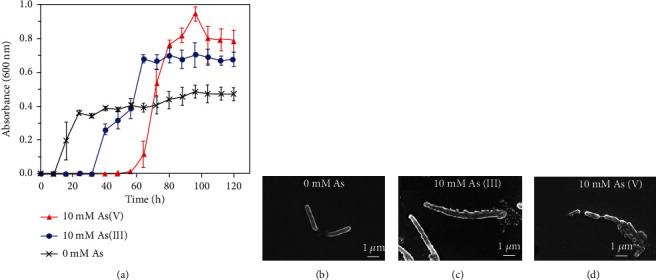
Effect of dissolved As(V) and As(III) on the (a) growth and (b, c, and d) morphology of strain IITK SM2. Bacteria were inoculated in 10 mM glucose-containing minimum salt media (MSM) and supplemented with either 10 mM As(V), or 10 mM As(III), or no As. Error bars correspond to standard deviations of the means from triplicate experiments. No background As(V) and As(III) was detected in MSM.

The growth of IITK SM2 was found to be more in the presence of arsenic as compared to As-free condition in minimum salt media (MSM) supplemented with glucose (Figures S2 and 2a). The lag phase of this strain varied with the type of As (III versus V, Figure S2) stress provided. For the comparative growth profile study investigated for 0 mM As, 10 mM As(V) and 10 mM As(III) conditions, the shortest lag phase was observed in the absence of any As (8 h), followed by increasing lag phases in the presence of 10 mM As(III) (32 h) and 10 mM As(V) (48 h) (Figure 2(a)). Logarithmic growth was observed between 8-24 h, 32-60 h, and 56-96 h for As-free, 10 mM As(V)-containing and 10 mM As(III)-containing conditions, respectively. The maximum biomass was observed in the presence of As(V) followed by As(III) and As-free conditions (Figure 2(a)). The growth rate (*k*) and doubling time (DT) observed in the absence of As was 0.08 h^−1^ and 8.9 h, respectively. In As-stressed conditions, DT increased to 10.5 h and 17.4 h in the systems containing 10 mM As(V) [*k* = 0.07 h^−1^] and 10 mM As(III) [*k* = 0.04 h^−1^], respectively. Although the addition of dissolved As(V) and As(III) retarded the growth rate, no inhibitory effect of arsenic on the growth of this strain was observed at 10 mM of As dosage. On the contrary, higher biomass was obtained in As-stressed conditions suggesting that the isolate metabolizes As and obtains energy for its growth [74, 75]. Furthermore, more growth in the presence of As(V) as compared to As(III) indicated that strain IITK SM2 possibly had a mechanism to effectively metabolize As(V) relative to As(III).

The IITK SM2 strain was rod-shaped as confirmed by SEM analysis (Figures 2(b)–2(d)). The average length of a bacterial cell in the absence of dissolved As was 2.8 ± 0.6 *μ*m (Figure 2(b)). However, in the presence of As(III), the length of bacteria increased to 5.6 ± 2.2 *μ*m, which suggested that As(III) induced stress condition to this isolate (Figure 2(c)) that resulted in filamentation [76]. This elongation indicated that cell division might be affected due to As(III) stress. However, in the presence of As(V), this strain was found to be clustered together, with no significant change in the cell size (2.0 ± 0.6 *μ*m) as compared to that of the As-free condition (Figure 2(d)).

#### 3.2.4. Redox Transformation of Arsenic by IITK SM2

**Figure 3:**
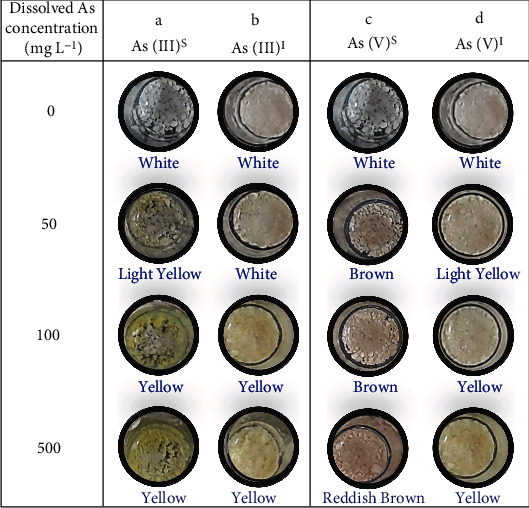
Redox transformation capability of strain IITK SM2 qualitatively confirmed with silver nitrate test. Isolates were cultured in minimum salt media, and 10 mM glucose was used as the carbon source. Color precipitates were obtained by adding Tris-HCl (0.1 M; pH = 7.4) and AgNO_3_. White precipitates were developed in the absence of any arsenic due to the formation of AgCl. Yellow [Ag_3_AsO_3(s)_] and brown precipitates [Ag_3_AsO_4(s)_] represent the presence of As(III) and As(V), respectively. Whereas As(III)^s^ and As(V)^s^ represent systems without any bacterial inoculation, The As(III)^I^ and As(V)^I^ represent systems with bacterial inoculation [1% (*v*/*v*); OD~ 1]. No background As(V) and As(III) was detected in MSM.

Qualitative silver nitrate assay indicated that strain IITK SM2 was an As(V) reducer (Figure 3). The formation of yellow- and brown-colored precipitates was observed with standard salts of As(III) [*As(III)^s^*; Figure 3a] and As(V) [*As(V)^s^*; Figure 3c], respectively, possibly due to precipitation of Ag_3_As^III^O_3(s)_ and Ag_3_As^V^O_4(s)_ [44]. However, in the presence of strain IITK SM2, the yellow color in precipitated solids was retained under As(III)-stressed conditions [*As(III)^I^*; Figure 3(b)], but brown precipitates were not observed for As(V) conditions [*As(V)^I^*; Figure 3(d)]. In fact, the precipitated solids were increasingly yellow with increasing As(V) concentrations. Further, measurements of As_T_ by ICP-MS, and of As(V) and As(III) by IC-ICP-MS, before and after the reaction with IITK SM2, confirmed these qualitative results. A complete reduction of As(V) to As(III) was observed in As(V)-amended conditions, whereas no change was observed in the conditions initially containing As(III) (Table S6 of the Supporting Information). Overall, results indicate that the isolate can mediate As(V) reduction to As(III), but not vice-versa. The presence of arsenate reducers, like *C. youngae* IITK SM2, in the middle Gangetic plain groundwater, could be the reason for significant concentrations of dissolved As(III) in oxidizing conditions for arsenic speciation.

### 3.3. Genes Identified in Arsenic Metabolism of IITK SM2

#### 3.3.1. Whole-Genome Sequencing (WGS) and Comparison with Other Organisms

To identify the species of IITKSM2, WGS was performed. The mean fragment size of PCR-enriched library of g-DNA of the strain was found to be 564 bp with a concentration of 14.1 ng *μ*L^−1^. In total, 2,672,974 bp raw reads were obtained for g-DNA, whereas total reads that survived after trimming and filtering were 1,778,915. The final genome size was found to be 4,857,938 bp with a guanine-cytosine (GC) content of 51.70% with N_50_ value of 438,827 bp. The respective assembly length was ~4.8 Mbp. The NCBI-PGAP annotation showed that the g-DNA contained a total of 4725 genes, which were associated with 4553 coding sequences (CDs) and 66 tRNA, 6 rRNA, and 9 noncoding RNA (ncRNA) sequences. Besides, 91 pseudogenes were also present.

Distance-based phylogeny developed by considering *recN* sequence indicated that IITK SM2 was clustered with C. *youngae* NCTC 13708^T^ and *C. pasteuri* NCTC UMH17^T^ (Figure S4 of the Supporting Information). However, the draft sequence of g-DNA of the strain showed the maximum average nucleotide identity (ANI) of 86.7% with *Citrobacter youngae* NCTC 13708^T^, followed by 62.43% ANI with *Citrobacter freundii* FDAARGOS 549^T^. Moreover, DNA-DNA hybridization (dDDH) analysis suggested that the intergenomic distance of our isolate was the closest to *C. youngae* NCTC 13708^T^ (85.8%), followed by *C. youngae* CCUG 30791^T^ (83.8%). As the proposed cut-off for species delineation is 70% [77, 78], these distances confirm that IITK SM2 belongs to a strain of *C. youngae* (Table 3). Furthermore, the difference in GC content between IITK SM2 and type strains of *C. youngae* was ≤0.1%, which confirms that the isolate is a strain of *C. youngae*.

**Figure 4:**
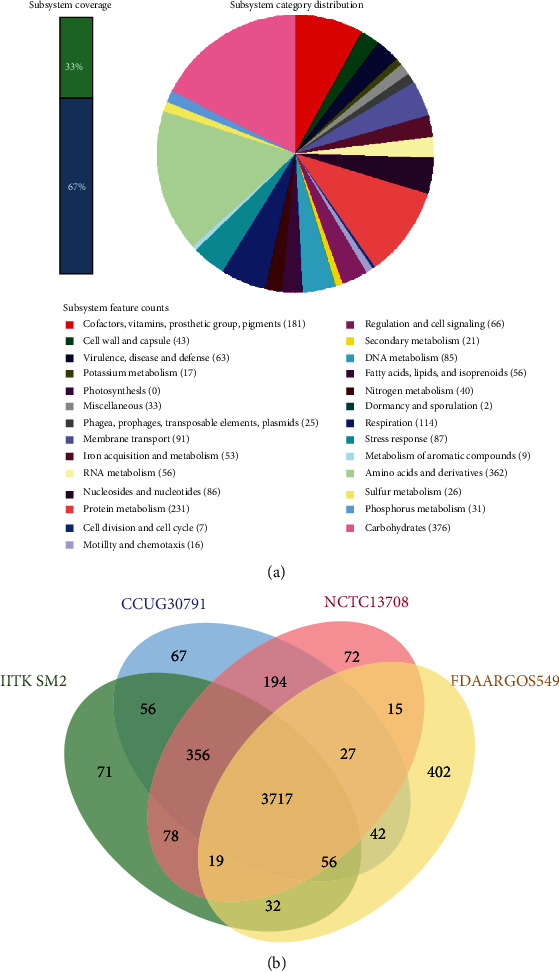
Distribution of subsystem features (a) and orthologous protein clusters (b) in the genome of *Citrobacter youngae* strain IITK SM2 of subsystem features was performed using the rapid annotations using subsystems technology (RAST) server, where RASTtk annotation scheme was used [57]. The Venn diagram of the clustering of proteins based on the coding sequences (CDs) was constructed using the whole genome sequences of isolate IITK SM2 and of strains of *Citrobacter youngae* (CCUG 30791 and NCTC 13708) and *Citrobacter freundii* (FDAARGOS 549).

Although IITK SM2 belongs to *C. youngae*, it is different from other *C. youngae* strains as suggested by the comparison of subsystem features and gene clustering of different *Citrobacter* species. The coverage of subsystem features in IITK SM2 and comparison of counts of each feature in different *Citrobacter* species using Rapid Annotations using Subsystems Technology (RAST) server suggested that IITK SM2 was different from the other type strains of *C. youngae* (Figure 4 and Table 4). Subsystem features grouped under virulence, disease, defense (F3), phages, prophages, transposable elements, plasmids (F7), and membrane transporter (F8) were much higher in strain IITK SM2 as compared to other type strains of *C. youngae* [NCTC 13708^T^ and CCUG 30791^T^; Table 4]. On the contrary, much lesser feature counts of motility and chemotaxis (F14) and protein metabolism (F12) were observed in IITK SM2. Furthermore, a Venn diagram of protein clustering of strain IITK SM2 with closely related *Citrobacter* strains, NCTC 13708, CCUG 30791, and FDAARGOS 549, indicated a total of 4653 protein clusters (Figure 4(b)). Of these, 4590 orthologous clusters that contained at least two strains and 3686 were single-copy clusters. Although IITK SM2 shared 3718 orthologous protein clusters with the other three strains, maximum clusters (4184) were shared with CCUG 30791. Most of the unique orthologous clusters identified in IITK SM2 represented proteins with unknown functions. However, two protein clusters were identified as the IS11595 family transposase and metal binding proteins. These analyses confirmed that our isolate is a novel strain of *Citrobacter youngae* and was named *Citrobacter youngae* IITK SM2.

#### 3.3.2. Presence of Arsenic-Resistant Genes

**Figure 5:**
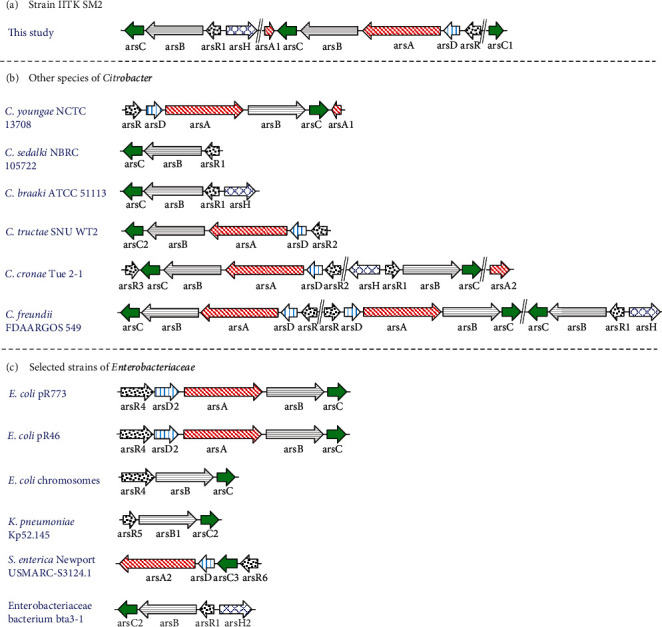
Comparison of arsenic-resistant genes identified in strain IITK SM2 with genes of other reference organisms: arsenic operon system in (a) strain IITK SM2, (b) other *Citrobacter* species, and (c) selected strains of *Enterobacteriaceae* a genus other than *Citrobacter*. The whole-genome sequence of each strain was considered for representing this schematic. Numbers appended after the name of some As-resistant genes indicate the difference in nucleotides. The gene bank or accession number of *Citrobacter* species used in this analysis is shown in Table S4 of the Supporting Information.

From the draft genome of isolate, *Citrobacter youngae* IITK SM2 As-resistant genes were identified (Figure 5(a)). These genes belongs to the *ars* operon system [33, 35, 79]. Genes corresponding to *ars*C, *ars*B, *ars*A, *ars*D, *ars*R, and *ars*H were present in the g-DNA of this isolate. These genes have specific functions. The arsenate reductase *ars*C, which encodes a protein of smaller molecular weight (13-15 kDa), belongs to the thioredoxin superfamily and mediates the reduction of As(V) to As(III) in the cytoplasm [79, 80]. The *ars* operon further contains an efflux pump (*ars*B) specific to arsenic, which encodes arsenite permease and extrudes As(III) out of the cell [81]. Resistance to As(V) and As(III) is provided by the expressions of *ars*C and *ars*B genes, which are controlled by a transcriptional repressor, *ars*R [80, 82]. The presence of these As-resistant genes could be the possible reason of such high MICs of As(V) and As(III) observed in strain IITK SM2. In addition to these genes, As-operon also contains *ars*D and *ars*A. It is known that *ars*D exhibits a weak As(III)-responsive transcriptional repressor activity [83], and the gene *ars*A provides higher resistance to elevated levels of As(III) by encoding intracellular ATPase, which forms a dimer with *ars*B [79].

The arsenic-resistant gene *ars*H is a NADPH-dependent flavin mononucleotide reductase [84] and was also identified in the g-DNA of IITK SM2. It was reported that the presence of an *arsH* gene increased resistance to inorganic As species (^i^As^V/III^) in some bacteria [85, 86]. However, some studies proved that neither overexpression nor mutation of *ars*H protein provided resistance to inorganic As in *Thiobacillus ferrooxidans* [87] and cyanobacterium *Synechocystis* sp. *PCC 6803* [88]. The exact function of the *ars*H gene remains unclear. Recently, a study showed that *ars*H detoxified organoarsenic compounds like MMA(III) and aromatic arsenic species by their oxidation to MMA(V) [89]. These As-resistant genes were arranged in three distinct ways, which contained (1) *ars*CBRH, (2) *ars*ACBADR, and (3) an independent *ars*C gene in strain IITK SM2 (Figure 5(a)). It is possible that the presence of these genes might have a role in high arsenic resistance exhibited by IITK SM2.

#### 3.3.3. Comparison of As-Resistant Genes in Strain IITK SM2 with Genes in Reference Organisms

The distribution of *ars* genes identified in the isolate differed from other type strains of *Enterobacteriaceae and especially of Citrobacter species* (Figure 5). *C. youngae* NCTC 13708 was the closest to IITK SM2, which contained the unique six gene operon (*ars*ACBADR) but was regulated in the opposite direction compared to IITK SM2 (Figure 5(b)). Such a sextet gene cluster was neither observed for any other *Citrobacter* species nor for any mentioned pioneer strains of the *Enterobacteriaceae* family. Besides this, operon IITK SM2 contained the *ars*CBRH operon and an independent *ars*C gene (Figure 5(a)), which were absent in NCTC 13708. These differences suggest that our isolate could be even more effective in arsenic resistance than NCTC 13708. Although *ars*CBRH operon was present in other *Citrobacter* species, such as *C. braakii* ATCC 51113^T^, *C. freundii* FDAARGOS 549^T^, and strain bta3-1^T^, the independent *arsC* gene was not identified in any of the chosen reference strains (Figures 5(b) and 5(c)). Earlier studies have referred to such proteins as “fusion proteins,” which if functional could provide evolutionary advantage in sensing and/or detoxifying As(III) in the environment [90, 91]. Other reference chromosomes or plasmids mostly contain the five-gene *ars*CBADR operon identified in *C. tructae* SNU WT2^T^, *C. freundii* FDAARGOS 549^T^ and *E. coli* (R773 and R46), or the three-gene *ars*RBC operon present in *C. sedalki* NBRC 105722^T^, *K. pneumoniae* Kp52.145^T^_,_ and *E. coli* chromosomes. An exception in the arrangement of As metabolizing genes in *C. cronae* Tue2-1^T^ was observed, where two transacting repressors were identified (*ars*RCBDAR). These *ars*CBADR and *ars*RBC were not identified in strain IITK SM2. Even though a dDDH value of ~84% suggested that the other strain of *C. youngae*, CCUG 30791, was closer to IITK SM2, no As-metabolizing genes were identified in this type strain. The identified unique arrangement of *ars* genes in this isolate was consistent with the possibility that IITK SM2 is a novel As-resistant strain of *C. youngae.*

#### 3.3.4. Presence of Three Arsenate-Reductase (arsC) Genes

**Figure 6:**
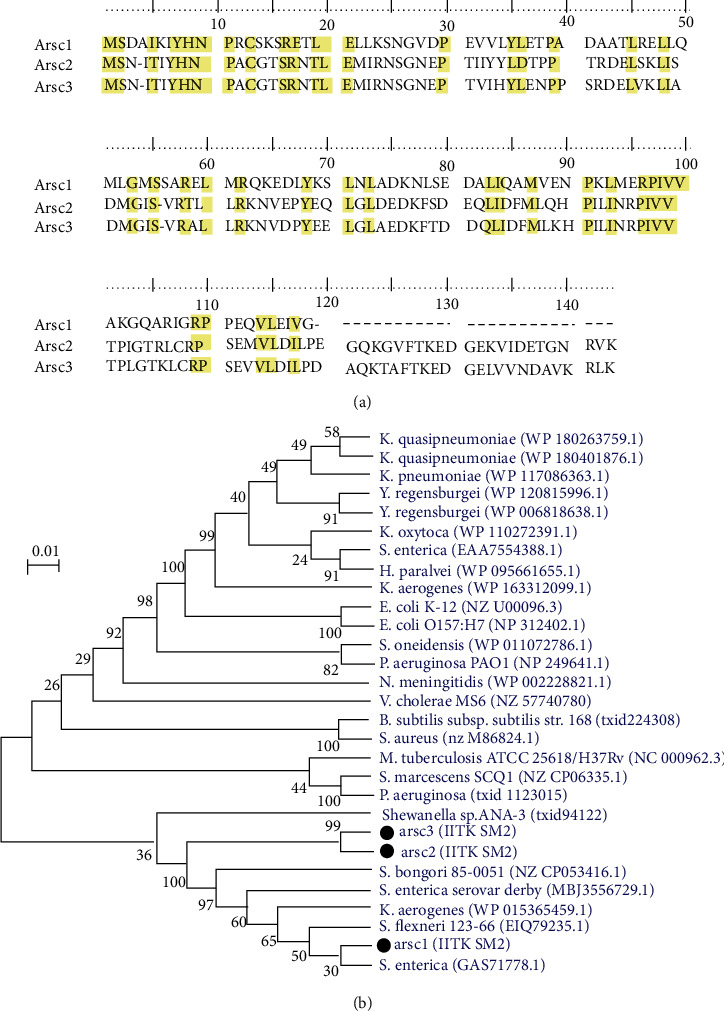
Multiple alignment (a) and phylogeny (b) of arsenate-reductase gene (*ars*C) sequences identified in the strain *Citrobacter youngae* IITK SM2. Multiple alignments of *ars*C sequences (*arsC*1, *arsC*2, and *arsC*3) of IITK SM2 were performed using CLUSTAL_W. Nucleotides conserved in all the three *ars*C genes are highlighted in yellow. The neighbor-joining method [57] was used for developing distance-based phylogeny of *ars*C genes. For this phylogeny, *ars*C genes in strain IITK SM2 were compared with other As(V)-reducing organisms (excluding strains of *Citrobacter*). Percentage bootstrap values corresponding to 1000 replicates are shown next to the branches. The tree shown in (b) was drawn to scale.

The isolate IITK SM2 contained three different *arsC* genes designated as *ars*C1, *ars*C2, and *ars*C3 (Figure 6). The numbers of amino acids in these arsenate reductases were 119, 141, and 141, respectively. The alignment of their protein sequences suggested that 41 amino acids were completely conserved in these three genes (Figure 6(a)). Furthermore, the protein sequences of *ars*C2 and *ars*C3 showed ~83% similarity. The phylogenetic tree based on NJ method suggested that the smaller arsenate-reductase, *arsC*1, clustered with *S. enterica* (GAS71778.1), whereas *ars*C2 and *ars*C3 were not clustered with any *ars*C's of the selected bacterial strains. However, *ars*C2 and *ars*C3 were clustered together (Figure 6(b)). This analysis suggested that the smaller arsenate reductase in strain IITK SM2 might have evolved from Salmonella-type strains, whereas the larger *arsC*'s might be native of *Citrobacter*. The presence of these arsenate reductases along with *ars*B, *ars*A, *ars*D, *ars*R, and *ars*H in strain IITK SM2 could be responsible for its high arsenic resistance and for As(V) reduction to As(III) (Figure S5). Detailed mechanism and kinetics of microbially mediated arsenate reduction by this isolate would help to understand the role of IITK SM2 on arsenic speciation in groundwater.

## 4. Conclusions

The Gram-negative, rod-shaped facultative anaerobe bacterial strain IITK SM2 could survive under high concentrations of dissolved As and could reduce As(V) to As(III). Apart from dissolved As, this isolate also showed resistance to some of the other heavy metals found in groundwater, such as Fe(III), Mn(II), Zn(II), Ba(II), and Cr(VI). Enhanced bacterial growth was observed in the presence of dissolved As(III) and As(V), but the former was more toxic to the cells than the latter. The IITK SM2 is a novel strain of *Citrobacter youngae* and is different from other strains in terms of a number of critical subsystem features related to membrane transporters, virulence and defense, motility, protein metabolism, and phages, prophages, and transposable elements. The presence of As-metabolizing genes such as *ars*C, *ars*B, *ars*A, *ars*D, *ars*R, and *ars*H were identified in the genome of this strain. A unique clustering of As-resistant genes was also found in the g-DNA of IITK SM2 as (1) *ars*CBRH, (2) *ars*ACBADR, and (3) an independent *ars*C gene, which was not observed in any other *Citrobacter* species and selected strains of *Enterobacteriaceae* family. Furthermore, two different varieties of *ars*C genes were identified, where one As(V)-reductase gene might have evolved from *Salmonella* and the other may have been native to *Citrobacter.* The information presented in this study could contribute to the mechanistic understanding of the biogeochemical processes that control elevated arsenic prevalence in groundwater, which could help in developing long term in situ mobilization techniques for As-remediation.

## Figures and Tables

**Table 1:** Geochemical parameters of groundwater samples collected from the study site in Kanpur, India.

Parameter	Units	As-polluted groundwater	As-unpolluted groundwater
*(i) General water quality parameters*			
pH	—	7.11	7.32
Measured redox potential (*E*_*H*_^*m*^)	mV	248	269
Calculated redox potential (*E*_*H*_^*c*^)	mV	112	n.a.
Temperature	°C	25.9	28.2
Conductivity	*μ*S cm^−1^	4021	1023
Alkalinity	mg L^−1^ as CaCO_3_	600	550
Estimated depth of the aquifer	m	12.2	30
Total organic carbon	mg L^−1^ as C	40	11
Dissolved inorganic carbon	mg L^−1^ as C	1296	186

*(ii) Elemental concentrations*			
Total arsenic (As_T_)	*μ*g L^−1^	70	2
As(V)		43	bdl
As(III)		22	bdl
Mn		1671	16
U		27	22
Al		2	bdl
Cr		6	bdl
Fe		106	838
Ni		3	10
Cu		9	2
Zn		23	259
Ba		569	83
V		bdl	bdl
Co		1	bdl
Se		10	bdl
Mo		92	bdl

Ca	mg L^−1^	101.5	69
Na		638.2	206
Mg		88.9	48
K		604.7	16
P		4.7	bdl

bdl: below detection limit. n.a.: not applicable.

**Table 2:** Characterization of *Citrobacter youngae* IITK SM2 isolated from As-polluted groundwater of Baikunthpur, India.

Characteristics	Strain IITK SM2
(a) *General parameters*	
Gram staining test	Gram-negative
Shape	Rod
Cell size (*μ*m)	0.3–0.6 × 2.2–3.4
Pigmentation (in Lysogeny agar plate)	Yellow

(b) *Growth conditions*	
pH range	4–10
Optimum pH	7.25
Temperature range (°C)	15-45
Optimum temperature (°C)	30
NaCl tolerance (%; *w*/*v*)	0-6
Optimum NaCl tolerance (%; *w*/*v*)	1.5

(c) *Minimum inhibitory concentration*	
Dissolved As(III)	1,100 mg L^−1^ (~14.7 mM)
Dissolved As(V)	32,000 mg L^−1^ (~427.1 mM)
Dissolved Fe(III)	1,000 mg L^−1^ (~17.9 mM)
Dissolved Mn(II)	5,000 mg L^−1^ (~91.0 mM)
Dissolved Cr(VI)	10 mg L^−1^ (~0.2 mM)
Dissolved Ba(II)	1100 mg L^−1^ (~14.7 mM)
Dissolved Zn(II)	250 mg L^−1^ (~3.8 mM)

(d) Antibiotic resistance^*α*^	
Ampicillin	100 *μ*g L^−1^
Hygromycin	50 *μ*g L^−1^

(e) *Other specific parameters*	
Utilization of D-glucose	+
Catalase	+
Motility	+
Starch hydrolysis	—
G + C content (%)	51.7
Arsenic transformation capability	Reduces As(V) to As(III)

^*α*^No resistance to kanamycin (50 *μ*g L^−1^), chloramphenicol (25 *μ*g L^−1^), ciprofloxacin (20 *μ*g L^−1^), gentamycin (10 *μ*g L^−1^), and streptomycin (50 *μ*g L^−1^) was observed.

**Table 3:** Intergenomic distance of isolate “IITK SM2” with different strains of Citrobacter using digital DNA-DNA hybridization (dDDH) analysis. Recommended formula 2^*α*^ was used for estimating the intergenomic distance. Accession numbers of strains used in this analysis are detailed in Table S4 of the Supporting Information.

Type of strain	dDDH value (%)	GC content difference (%)
*Citrobacter youngae* NCTC 13708	85.8	0.10
*Citrobacter youngae* 30791	83.8	0.09
*Citrobacter pasteurii* CIP 55.13	60.7	0.10
*Citrobacter freundii* FDAARGOS 549	42.0	0.03
*Citrobacter freundii* NBRC 12681	41.9	0.04
*Citrobacter portucalensis* A60	39.2	0.31
*Citrobacter europaeus* 97/79	39.0	0.18
*Citrobacter braakii* ATCC 51113	38.9	0.20
*Citrobacter cronae* Tue2-1	36.7	0.71
*Citrobacter werkmanii* NBRC 105721	36.4	0.37
*Citrobacter tructae* SNU WT2	32.9	0.25
*Citrobacter koseri* NCTC 10786	26.4	2.10
*Citrobacter amalonaticus* NCTC 10805	25.2	1.73
*Citrobacter rodentium* NBRC 105723	24.7	2.94
*Citrobacter sedlakii* NBRC 105722	24.4	3.01

^*α*^[51].

**Table 4:** Comparison of subsystem features in different *Citrobacter* species using rapid annotations using subsystems technology (RAST) server. The annotation scheme used was RASTtk [57]. Accession numbers of different type strains are mentioned in Table S4 of the Supporting Information.

S.no.	Parameters	Number of parameters/features								
		*C. youngae* IITK SM2*^a^*	*C. youngae* NCTC 13708	*C. youngae* CCUG 30791	*C. Freundii* FDAARGOS 549	*C. Sedlakii* NBRC 105722	*C. Cronae* Tue2-1	*C. Tructae* SNU WT2	*C.* Braakii ATCC 51113	*C. Rodentium* NBRC 105729
*General parameters*										
G1	Total number of features	4725	4855	4872	4856	4507	5608	4686	5717	5297
G2	Number of coding sequences	4644	4747	4789	4747	4426	5514	4578	5619	5297
G3	Number of RNAs	81	108	83	109	81	94	108	98	70
G4	GC content	51.7	51.8	51.8	51.7	54.7	52.7	51.9	51.9	54.6

*Subsystem feature*										
F1	Cofactors, vitamins, prosthetic groups, and pigments	181	179	180	182	175	200	179	183	180
F2	Cell wall and capsule	43	44	44	43	41	44	46	47	42
F3	Virulence, disease, and defense	63	53	55	53	50	74	52	59	39
F4	Potassium metabolism	17	17	17	17	15	17	17	17	14
F5	Photosynthesis	0	0	0	0	0	0	0	0	0
F6	Miscellaneous	33	33	33	35	28	34	35	35	19
F7	Phages, prophages, transposable elements, and plasmids	25	7	10	14	5	42	15	26	13
F8	Membrane transport	91	70	72	74	77	86	67	106	79
F9	Iron acquisition and metabolism	53	53	54	46	27	37	39	42	26
F10	RNA metabolism	56	57	57	57	55	59	57	58	60
F11	Nucleosides and nucleotides	86	80	80	82	79	87	78	81	84
F12	Protein metabolism	231	253	236	262	255	250	210	247	198
F13	Cell division and cell cycle	7	7	7	7	7	7	7	7	7
F14	Motility and chemotaxis	16	101	97	15	101	98	96	100	89
F15	Regulation and cell signaling	66	65	68	61	54	88	63	68	51
F16	Secondary metabolism	21	21	21	23	4	21	21	22	5
F17	DNA metabolism	85	78	78	74	83	103	74	102	89
F18	Fatty acids, lipids, and isoprenoids	56	55	61	54	58	58	56	56	52
F19	Nitrogen metabolism	40	40	40	51	45	50	40	40	46
F20	Dormancy and sporulation	2	2	2	2	2	2	2	2	2
F21	Respiration	114	111	114	119	106	135	116	117	103
F22	Stress response	87	86	86	96	93	100	89	93	94
F23	Metabolism of aromatic compounds	9	9	9	20	35	17	6	13	34
F24	Amino acids and derivatives	362	364	368	385	331	399	349	369	336
F25	Sulfur metabolism	26	26	28	23	22	23	21	21	26
F26	Phosphorus metabolism	31	31	31	31	29	31	30	31	30
F27	Carbohydrates	376	387	378	411	367	425	387	394	396

*^a^* This study.

## Data Availability

The GenBank accession number for the 16S rRNA sequence is MZ477215. The accession number of the whole-genome shotgun project of *Citrobacter youngae* IITK SM2 registered at DDBJ/ENA/GenBank is JAGIYN000000000. In this study, the version described is JAGIYN000000000.1. Other data are included within the manuscript.

## Abbreviations

As: Arsenic
As(III): Arsenite
As(V): Arsenate
IGP: Indo-Gangetic plain
g-DNA: Genomic DNA
WGS: Whole-genome sequencing
LB: Lysogeny broth
GC: Guanine-cytosine
MSM: Minimum salt media
MIC: Minimum inhibitory concentration
MHB: Mueller Hinton broth
SEM: Scanning electron microscopy
ICP-MS: Inductively coupled plasma mass spectroscopy
IC-ICP-MS: Ion chromatography coupled with inductively coupled plasma mass spectroscopy
NCBI: National Center for Biotechnology Information
BLAST: Basic local alignment search tool
TOC: Total organic carbon
DIC: Dissolved inorganic carbon
dDDH: Digital DNA-DNA hybridization
TYGS: Type (strain) genome server
NJ: Neighbor joining
PGAP: Prokaryotic genome annotation pipeline
OM: Organic matter
MMA(III): Monomethylated arsenous acid
MMA(V): Monomethylated arsenic acid
RAST: Rapid annotations using subsystems technology.

## Supplementary Materials

Supplementary Materials A Supporting Information file of 24 pages containing six sections, five figures, and six tables is available online. This file contains details of sample collections, analysis techniques, experimental procedure, phylogenetic analysis based on *recN* sequence, map of sampling location, composition of media used, results from biochemical experiments, details of chemicals and media used, method detection limits of various techniques used, and comparison of strain IITK SM2 with other arsenate-reducers identified in the literature.
