# Supplementary material for: Identification and Genome Analysis of an Arsenic-Metabolizing Strain of Citrobacter youngae IITK SM2 in Middle Indo-Gangetic Plain Groundwater
Source: Biomed Res Int. 2022 Mar 10;2022:6384742. doi: 10.1155/2022/6384742 (PMC8930248; doi:10.1155/2022/6384742)
Supplement: Supplementary Materials — A Supporting Information file of 24 pages containing six sections, five figures, and six tables is available online. This file contains details of sample collections, analysis techniques, experimental procedure, phylogenetic analysis based on recN sequence, map of sampling location, composition of media used, results from biochemical experiments, details of chemicals and media used, method detection limits of various techniques used, and comparison of strain IITK SM2 with other arsenate-reducers identified in the literature. [file 6384742.f1.docx]

# Supporting Information

**Identification and Genome Analysis of an Arsenic-Metabolizing Strain of *Citrobacter youngae* IITK SM2 in Middle Indo-Gangetic Plain Groundwater**

Akshat Verma^1,2^, Prem Anand Murugan^3,4^, Hariharan Vedi Chinnasamy^3,4^, Abhas Singh^1,2*^,

Saravanan Matheshwaran^3,4,5*^

*^1^Department of Civil Engineering, ^2^Environmental Geochemistry Laboratory, Centre for Environmental Science and Engineering, ^3^Department of Biological Sciences and Bioengineering, ^4^Environmental Microbiology Laboratory, Center for Environmental Science and Engineering,  ^5^Mehta Family Center for Engineering and Medicine*

Indian Institute of Technology Kanpur, India

Revised manuscript submitted to *BioMed Research International*

**This document contains a total of 24 pages containing 6sections, 5 figures and 6 tables.**

**^*^ Details of corresponding authors:**

Dr. Saravanan Matheshwaran, phone: +91-512 679 4066; fax: +91-512 679 4010, email: saran@iitk.ac.in

Dr. Abhas Singh, phone: +91-512 679 7665; fax: +91-512 679 4066, email: abhas@iitk.ac.in

| **Table of Contents** | | |  |
| --- | --- | --- | --- |
| ***List of sections*** | | | |
|  | Section S1 | Calculated redox potential ($E_{H}^{m})$of As-polluted groundwater sample……….. | 3 |
|  | Section S2 | Staining, optimum growth conditions and antibiotic resistance……………. | 4 |
|  | Section S3 | Sample preparation for SEM analysis…………………………………….... | 5 |
|  | Section S4 | Determination of minimum inhibitory concentration (MIC)....................….... | 5 |
|  | Section S5 | Other details of qualitative As-redox transformation capacity………….……. | 6 |
|  | Section S6 | Molecular characterization: Isolation of genomic DNA and 16S rRNA sequencing………………………………………………………………….… | 6 |
| ***List of figures*** | | | |
|  | Figure S1 | Sampling locations at Baikunthpur, Kanpur Nagar, India, where the  groundwaters were collected to isolate of arsenic-resistant bacteria**.** ………………… | 7 |
|  | Figure S2 | Effect of dissolved As(V) and As(III) on the growth of strain IITK SM2. | 8 |
|  | Figure S3 | Optimum growth conditions for the growth of strain *IITK SM2*………………….… | 9 |
|  | Figure S4 | Neighbor-joining tree based on *recN* sequencing, showing the position of  strain IITK SM2 with other strains of genus *Citrobacter……………………………* | 10 |
|  | Figure S5 | Schematics of As(V) reduction to As(III) by a novel stain *of Citrobacter youngae* IITK SM2……………………………………………………………… | 11 |
| ***List of tables*** | | |  |
|  | Table S1 | Reagents and standards used in the study for various analyses …………….... | 12 |
|  | Table S2 | Method detection limits of various techniques for the measurement of different parameters.…...………………………………………………….….. | 14 |
|  | Table S3 | Composition of minimum salt media (MSM)………………..……………….. | 15 |
|  | Table S4 | Overview of different type strains and corresponding accession/Gen bank numbers used in this study……………………………………………………. | 16 |
|  | Table S5 | Comparison of arsenate-reducing, Gram-negative, rod-shaped bacteria from existing literature………..……………………………………………………. | 18 |
|  | Table S6. | Dissolved arsenic concentration in the absence and presence of *Citrobacter youngae* IITK SM2……………………………………………………………. | 20 |

**SUPPORTING SECTIONS**

**Section S1. Calculated redox potential (**$\boldsymbol{E}_{\boldsymbol{H}}^{\boldsymbol{m}}\boldsymbol{)}$**of As-polluted groundwater sample**

Following aqueous reactions and stability constants were used for this calculation:

| 1. ***Arsenate and arsenite acid-base*** | | |
| --- | --- | --- |
| H_3_AsO_4_ = H_2_AsO_4_^-^ +H^+^ | Log K = -2.25 [1] | (S1) |
| H_2_AsO_4_^-^ = HAsO_4_^2-^ +H^+^ | Log K = -6.98 [1] | (S2) |
| HAsO_4_^2-^ = AsO_4_^3-^ +H^+^ | Log K = -11.58 [1] | (S3) |
| H_3_AsO_3_ = H_2_AsO_3_^-^ +H^+^ | Log K = -9.24 [1] | (S4) |
| H_2_AsO_3_^-^ = HAsO_3_^2-^ +H^+^ | Log K = -14.10 [1] | (S5) |
| HAsO_3_^2-^ = AsO_3_^3-^ +H^+^ | Log K = -15.00 [1] | (S6) |
| ***Associated redox*:** | | |
| AsO_4_^-3^ +2H^+^ +2e^-^↔ AsO_3_^-3^+H_2_O | Log K = 5.29 [1] | (S7) |
| O_2_(g) +4H^+^+4e^-^ ↔ 2H_2_O | Log K = 83.12 [1] | (S8) |
| 2H^+^+2e^-^ ↔ H_2_(g) | Log K = 0.00 [1] | (S9) |
| AsO_4_^-3^ +2H^+^ +2e^-^+↔ AsO_3_^-3^+H_2_O | logK = 43.63 [2] | (S10) |

In circumneutral pH range (pH = 6-8), the redox reaction listed as eq. S10 will be used. Using the Nernst equation, the measured redox potential ($E_{H}^{m})$of eq. S10 is given by

$E_{H}^{m} = E_{H}^{0}- \frac{0.059}{n}\times log\left( \frac{\left\{ H_{3}\mathrm{As}O_{3\left( \mathrm{aq} \right)} \right\} \times\left\{ H_{2}O \right\}}{\left\{ \mathrm{AsO}_{4}^{3-} \right\} \times\left\{ H^{+} \right\}^{5}} \right)$ [3] (S11)

where,

$E_{H}^{0}$ – standard redox potential. This is related by standard state free energy (ΔG^0^) and solubility constant (K) of the above reaction as: $\Delta G^{0}= -nFE_{H}^{0}= -RTlnK$ (S12)

n- number of electrons transferred in the redox reaction (= 2, here), *{H^+^} = 10^-pH^,* F is Faraday’s constant = 96,485 C mol^-1^, and R is the universal gas constant = 8.314 J mol^-1^ K^-1^.

Using eqs. S10 and S12, $E_{H}^{0}$ = 1.96 V (at T = 273 K)……………………….…………….(S13)

Again, $\left\{ \mathrm{As}O_{4}^{3-} \right\}= \frac{\mathrm{TOTAs}\left( V \right)\times K_{a1}\times K_{a2}{\times K}_{a3}}{{{(10}^{-pH})}^{3}+K_{a1}{{(10}^{-pH})}^{2}+K_{a1}K_{a2}{(10}^{-pH})+K_{a1}K_{a2}K_{a3}}$ (S14)

$\Rightarrow\left\{ H_{3}\mathrm{As}O_{3\left( \mathrm{aq} \right)} \right\}= \frac{\mathrm{TOTAs}\left( \mathrm{III} \right)\times{{(10}^{-pH})}^{3}}{{{(10}^{-pH})}^{3}+K_{b1}{{(10}^{-pH})}^{2}+K_{b1}K_{b2}{(10}^{-pH})+K_{b1}K_{b2}K_{b3}}$ (S15)

where, $K_{a1}$, $K_{a2}$ and $K_{a3}$ and $K_{b1}$, $K_{b2}$ and $K_{b3}$ are the dissociation constants of arsenic acid ($H_{3}\mathrm{As}O_{4\left( \mathrm{aq} \right)}$) and arsenous acid ($H_{3}\mathrm{As}O_{3\left( \mathrm{aq} \right)})$, respectively, {Nordstrom, 2003 #125;Nordstrom, 2003 #125}listed in eqs S1-S6.

$E_{H}^{m}$ can be computed using equations S11, S13, S14 and S15.

## Section S2. Morphological, physiological, and biochemical characterization of strain IITK SM2

To identify the bacterial group, of strain IITK SM2 Gram-staining was performed, and the morphology of the strain was assessed with optical microscopy [5, 6].

For the determination of optimum growth conditions of strain IIK SM2, tests related to specific environmental parameters were performed. Optimum pH for the growth was determined by inoculating this isolate in LB over pH 2-12 at 30^o^C. Growth at different temperatures (4-50 ^o^C) was assessed for 24 h at the optimum pH (pH_opt_= 7.25) determined above. The salt tolerance limits for growth were observed in a rich media that consisted of 1 % tryptone, 0.5% yeast extract, and different concentrations of NaCl (0-8 %, w/v) at the optimum pH and temperature (Temp_opt_ = 30 ºC) identified above. Growth under anaerobic conditions was investigated in an anaerobic chamber (Glove box; GP Campus; Jacomex; O_2_< 0.2 ppm).

Specific biochemical parameters were also tested for this strain. For the utilization of D‑glucose IITK SM2 was inoculated in a minimum salt media (MSM) supplemented with 10 mM glucose (Table S3). To test for catalase activity, bubble production was assessed by the addition of 3 % (v/v) H_2_O_2_ [7]. For starch hydrolysis test, strain IITK SM2 was grown in starch agar for 48 h, to which iodine was added to check for the formation of a clear zone [7]. Motility was tested by observing the spread of the colonies in Lysogeny media with 0.5 % agar [8]. For this test, 3 µL of inoculum was placed at the center of a petri-plate that contained this semi-solid media, and the spread was monitored for 48 h. Resistance to antibiotics was tested by investigating the growth of strain IITK SM2 in LB in the presence of ampicillin (100 µg L^-1^), kanamycin (50 µg L^-1^), chloramphenicol (25 µg L^-1^), ciprofloxacin (20 µg L^‑1^), gentamycin (10 µg L^-1^), streptomycin (50 µg L^-1^), and hygromycin (50 µg L^-1^).

**Section S3. Sample preparation for SEM analysis**

For SEM-EDX analysis, IITK SM2 was inoculated in MSM with 10 mM glucose and supplemented with either no As, or 10 mM As(III), or 10 mM As(V). Each overnight grown bacterial sample was taken in a separate cover slip and diluted two times using As-fee MSM. The bacterial cells were washed using phosphate buffer saline (PBS). After this treatment, the cells were fixed with 2.5% glutaraldehyde (prepared in 1% PBS). After 30 min, the cells were rewashed with PBS. Subsequently, sequential dehydration was followed using increasing ethanol concentration (30%–100%) with incubation of 10 mins after addition of particular concentration of ethanol.

**Section S4. Determination of minimum inhibitory concentration (MIC)**

To determine MIC, IITK SM2 was initially inoculated in Mueller Hinton broth [MHB; [9]] without any chemical supplements. The culture was incubated overnight at 30 ^o^C and 120 rpm. This culture was used as an inoculum for further MIC experiments. Graded concentrations (0–50,000 mg L^-1^) of all heavy metals were prepared in MHB in 15 mL pre-autoclaved centrifuge tubes. The inoculum [1% (v/v); OD ~ 1] was transferred to these tubes. The inoculated metal containing solutions were then immediately transferred to respective 96‑well plates, where each column contained a fixed concentration of each metal. These plates were then incubated statically at 30 ^o^C for 16 h, followed by the addition of resazurin blue dye [~ 0.0015 %; [9, 10]]. The resazurin-supplemented well plates were observed for visible color change from blue to pink~~,~~ after incubation at 37 ^o^C for 3‑4 h. This color change occurred due to the reduction of resazurin to resofurin, which indicated the presence of aerobic-respiring bacteria that uptake O_2_. The MIC was identified as the lowest metal concentration at which the blue color was retained.

**Section S5. Details of qualitative As-redox transformation capacity**

For this experiment, bacterial cultures were grown in MSM supplemented with 10 mM glucose in the absence or presence of arsenic. No background dissolved arsenic was detected in MSM. After 3 d of incubation at 37 ^o^C, cultures were centrifuged, and supernatant (0.2 mL) was transferred to 24-well plates. About 0.8 mL of tris-HCl (0.1 M; pH ~ 7.4) and 1 mL of AgNO_3_ (0.1 M) were added to the wells. Precipitation of reddish-brown and bright-yellow-colored solids indicated the presence of Ag_3_As^V^O_4(s)_ and Ag_3_As^III^O_3(s)_, respectively.

**Section S6. Molecular characterization: Isolation of genomic DNA and 16S rRNA sequencing**

### Isolation of genomic DNA

A single colony of the strain was isolated by serial dilutions, followed by the streak-plate method [11]. After overnight incubation of isolate in 10 mL of LB at 37°C, genomic DNA (g-DNA) was purified via the phenol-chloroform extraction method [12]. Concentrations and quality of extracted DNA were determined using NanoDrop spectrophotometer and Qubit^®^ fluorometer.

### 16S rRNA sequencing

For 16S rRNA sequencing, polymerase chain reaction (PCR) primers—forward primer 5'‑AGA GTT TGA TCM TGG CTC AG‑3’ and reverse primer 5'‑TAC GGY TAC CTT GTT ACG ACT T 3'—were used for amplification.

**SUPPORTING FIGURES**


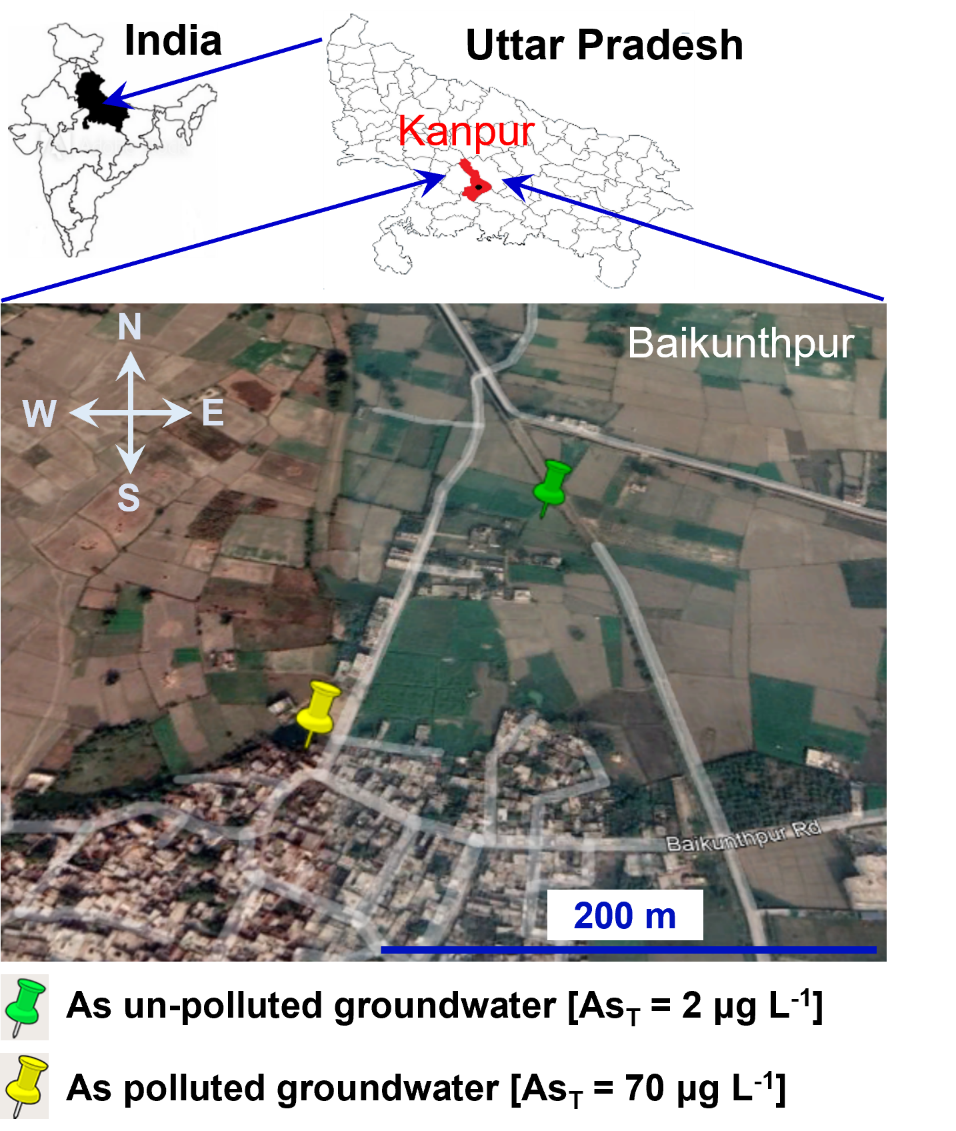


**Figure S1. Sampling locations at Baikunthpur, Kanpur Nagar, India, where the groundwaters were collected to isolate of arsenic-resistant bacteria.** The map was generated with Google Earth (<https://www.google.com/earth/versions/#download-pro>) containing measured GPS coordinates of locations (listed in Electronic Annex) centred around 26°33’47.3’’ N and 80°15’18.5’’E.


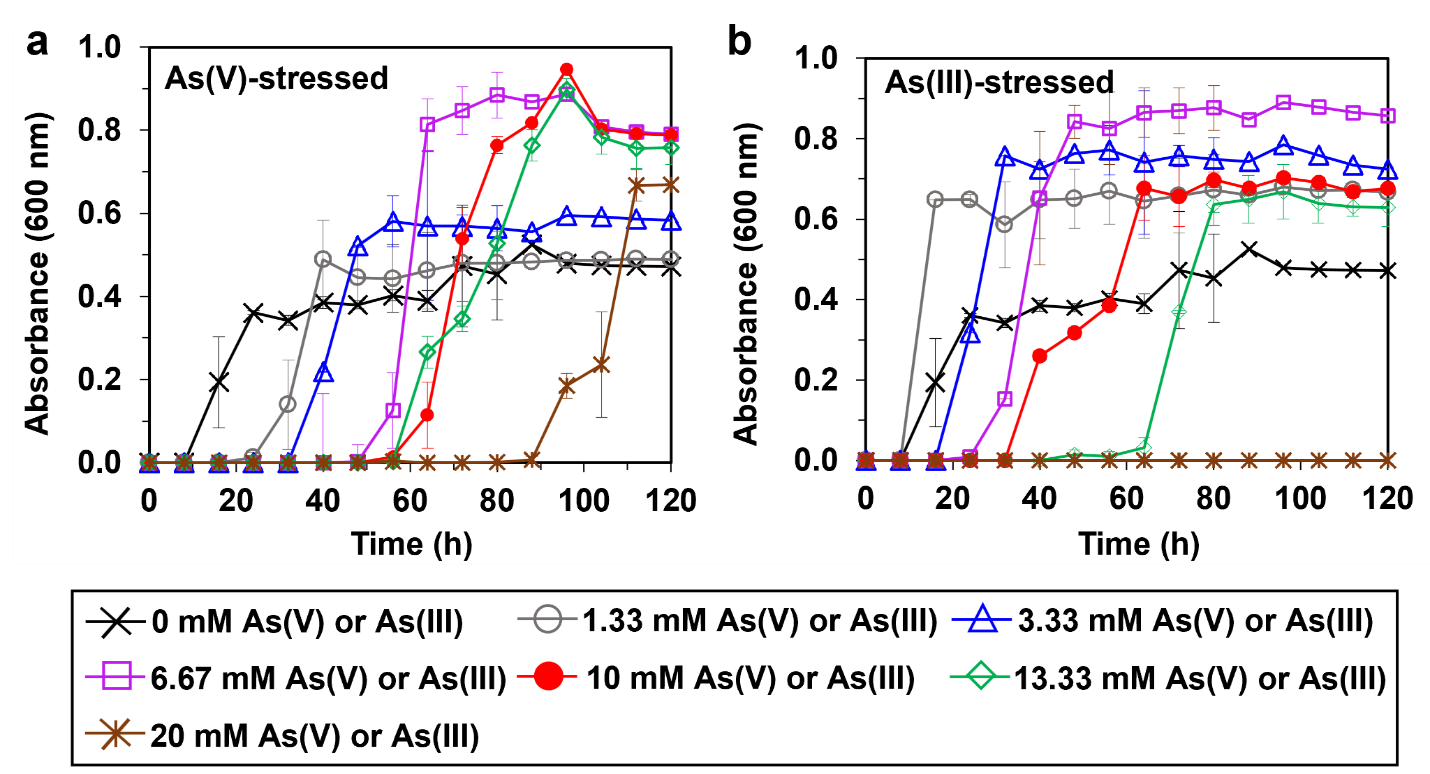


**Figure S2. Effect of dissolved (a) As(V) and (b) As(III) on the growth of strain IITK SM2.** Bacteria were inoculated in 10 mM glucose-containing minimum salt media (MSM) and supplemented with either 0-20 mM As(V), or 0-20 mM As(III. Error bars correspond to standard deviations of the means from triplicate experiments. No background As(V) and As(III) were detected in MSM.


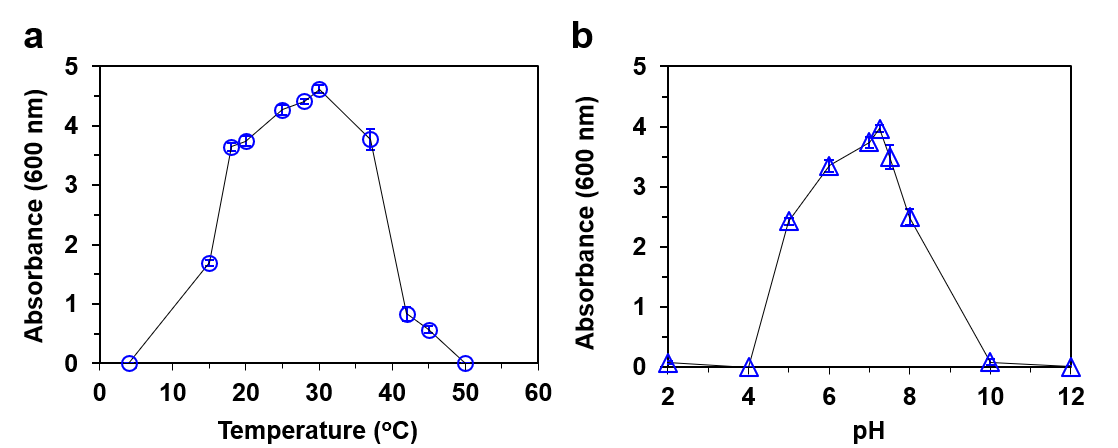


**Figure S3. Optimum growth conditions for the growth of strain *IITK SM2*.** Variation of maximum growth observed with the change in (a) temperature at a fixed optimum pH of ~7.25 and (b) pH at a fixed temperature of 30 ºC. The isolate was inoculated in Lysogeny broth (LB) broth. Error bars correspond to standard errors of the means of triplicate experiments.


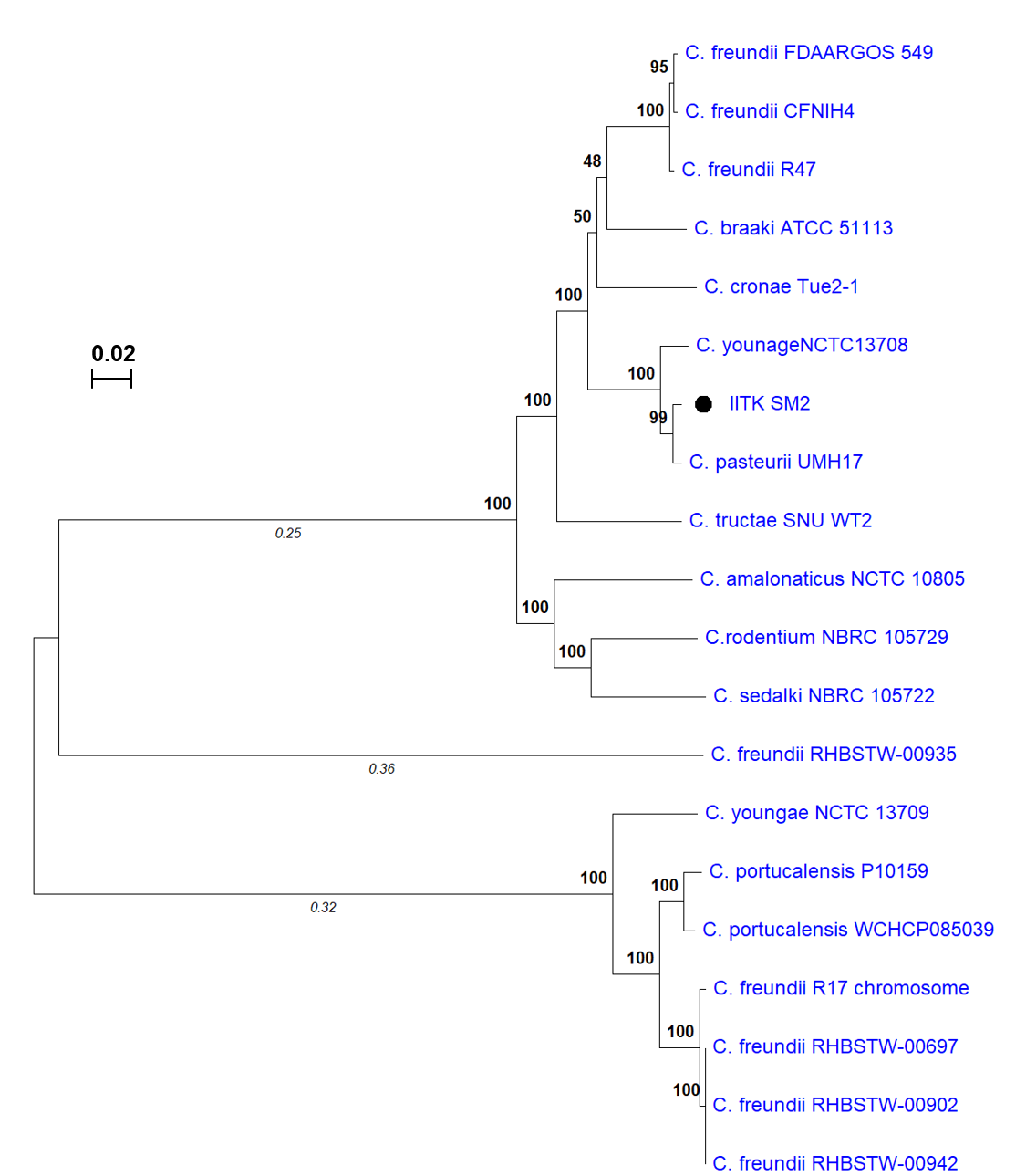


**Figure S4. Neighbor-joining tree based on *recN* sequences, showing the position of strain IITK SM2 with other strains of genus *Citrobacter***. Percentage bootstrap values corresponding to 1000 replicates are shown next to the branches in “bold”. Branch lengths are shown in “narrow italics” below each branch. The tree was drawn to scale


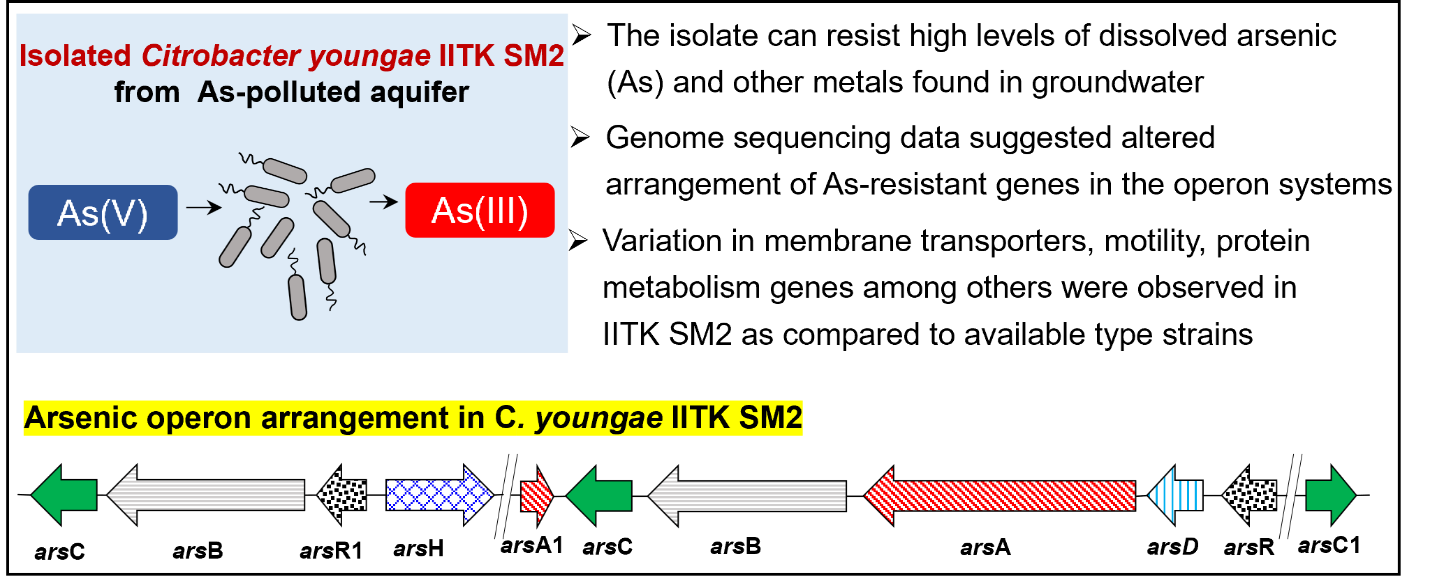


**Figure S5. Schematics of As(V) reduction to As(III) by a novel stain *of Citrobacter youngae* IITK SM2**

**SUPPORTING TABLES**

**Table S1. Reagents and standards used in the study for various analyses.**

| **S.No** | **Chemicals/ Standards** | **Linear Formula** | **% Purity** | **Grade/Certification** | **Manufacturer** |
| --- | --- | --- | --- | --- | --- |
| 1 | Sodium arsenate | Na_2_HAsO_4_·7H_2_O | $\geq$ 98.0 | ACS | Sigma-Aldrich |
| 2 | Sodium arsenite | NaAsO_2_ | 98.0 | ACS | MP Biomedicals |
| 3 | Sodium bromide | NaBr | $\geq$ 99.99 | ACS | Sigma-Aldrich |
| 4 | Nitric acid | HNO_3_ | 67-70.0 | Trace metal | Fisher Scientific |
| 5 | Multi-element standard | Ca, Fe, K, Mg, and Na in 5 % HNO_3_ | - | AA and ICP-OES calibration standards  (500 mg L^-1^) | Agilent Technologies |
| 6 | Multi-element standard | Be, Al, Ti, V, Cr, Mn, Co, Ni, Cu, Zn, As, Ag, Se, Th, Cd, Ba, Pb, U | - | AA and ICP-OES calibration standards  (100 mg L^-1^) | Agilent Technologies |
| 7 | Multi-element standard | Sn, Sb, Mo, Tl | - | AA and ICP-OES calibration standards  (500 mg L^-1^) | Agilent Technologies |
| 9 | As(V) standard | As(V) | - | ICP standard  (1000 mg L^-1^) | Sigma-Aldrich |
| 10 | As(III) standard | As(III) | - | ICP standard  (1000 mg L^-1^) | Sigma-Aldrich |
| 11 | Ammonium carbonate | (NH_4_)_2_CO_3_ | $\geq$30.0 NH_3_ basis | ACS | Alfa Aesar |
| 12 | Germanium | Ge | - | Standard for ICP | Alfa Aesar |
| 13 | Scandium | Sc | - | Standard for ICP | Sigma-Aldrich |
| 14 | Yttrium | Y | - | Standard for ICP | Sigma-Aldrich |
| 15 | Indium | In | - | Standard for ICP | Sigma-Aldrich |
| 16 | Bismuth | Bi | - | Standard for ICP | Alfa Aesar |
| 17 | Sodium chloride | NaCl | > 99.0 | ACS | Sigma-Aldrich |
| 18 | Potassium chloride | KCl | > 99.0 | ACS | Sigma-Aldrich |
| 19 | Magnesium chloride | MgCl_2_·6H_2_O | > 99.0 | ACS | Sigma-Aldrich |
| 20 | Calcium chloride | CaCl_2_·2H_2_O | > 99.0 | ACS | Sigma-Aldrich |
| 21 | Sodium sulphate | Na_2_SO_4_ | > 99.0 | ACS | Sigma-Aldrich |
| 22 | Di-sodium hydrogen phosphate | Na_2_HPO_4_ | > 99.0 | ACS | Sigma-Aldrich |
| 23 | Di-potassium hydrogen phosphate | K_2_HPO_4_ | > 99.0 | ACS | Sigma-Aldrich |
| 24 | Potassium phosphate monobasic | KH_2_PO_4_ | > 99.5 | Molecular biology | SRL Chemicals |
| 25 | Ammonium chloride | NH_4_Cl | $\geq$ 99.5 | ACS | Sigma-Aldrich |
| 26 | Tryptone Type-I | - | - | Molecular biology | Himedia |
| 27 | Yeast extract | - | - | Molecular biology | Himedia |
| 28 | Agar powder | - | - | Molecular biology | Himedia |
| 29 | Silver nitrate | AgNO_3_ | $\geq$ 99.0 | ACS | Sigma-Aldrich |
| 30 | Tris buffer | (HOCH_2_)_3_CNH_2_ | $\geq$ 99.9 | Molecular biology | SRL chemicals |
| 31 | Glucose | C_6_H_12_O_6_ | extrapure | Molecular biology | SRL chemicals |

**Table S2. Method detection limits of various techniques for the measurement of different parameters.**

| **Parameter** | **Technique** | **Detection limit** |
| --- | --- | --- |
| ***Instruments used for validation of JalKalp filter at laboratory*** | | |
| As_T_ (µg L^-1^) | ICP-MS | 0.1 |
| As(V) (µg L^-1^) | IC-IC-PMS | 0.5 |
| As(III) (µg L^-1^) | IC-IC-PMS | 0.5 |
| DIC (mg L^-1^) | TOC analyser | 0.1 |
| Absorbance | BioSpectrophotometer | 0.001 |

**Notes:**

As_T_ represents total dissolved arsenic and total dissolved iron.

Following notations were used for different techniques:

ICP-MS: Inductively coupled plasma mass spectrometry

IC-ICP-MS: Ion chromatography coupled with inductively coupled plasma mass spectrometry

TOC: Total organic carbon

**Table S3. Composition of minimum salt media (MSM)*.**

| **Chemical** | **Concentration (M)** |
| --- | --- |
| Tris buffer | 0.04953 |
| KCl | 0.02039 |
| NaCl | 0.08556 |
| NH_4_Cl | 0.01944 |
| Na_2_SO_4_ | 0.00282 |
| MgCl_2_.6H_2_O | 0.00098 |
| CaCl_2_.2H_2_O | 0.00020 |
| K_2_HPO4 | 0.00006 |
| KH_2_PO4 | 0.00007 |

**Glucose was added as 10 mM as the sole C-source*

**Table S4. Overview of different types of strains and corresponding accession/Gen bank numbers used in this study**

| **Strain** | **Accession No.** | **Strain** | **Accession No.** |
| --- | --- | --- | --- |
| **Whole genome sequences (WGS) of *Citrobacter* species** | | **16S rRNA sequence of *Citrobacter* species** | |
| *C. youngae* NCTC 13708 | NZ_UFWE01000006.1 | *C. koseri* LMG 5519 | NR_117751.1 |
| *C. youngae* CCUG 30791 | NZ_RPOI00000000.1 | *C. koseri* CIP 8287 | NR_118588.1 |
| *C. pasteurii* CIP 55.13 | NZ_CDHL01000039.1 | *C. koseri* *LMG 5519* | NR_118105.1 |
| *C. freundii* FDAARGOS 549 | NZ_CP033744.1 | *C. koseri CDC 8132-86* | NR_104890.1 |
| *C. freundii* NBRC 12681 | NZ_JMTA01000001.1 | *C. sedlakii* I 75 5519 | NR_028686.1 |
| *C. portucalensis* A60 | NZ_MVFY01000001.1 | *C. rodentium* DO 14784 | NR_028685.1 |
| *C. europaeus* 97/79 | NZ_FLYB03000035.1 | *C. farmeri* CDC 2991-81 | NR_024861.1 |
| *C. braakii* ATCC 51113 | NZ_NAEW00000000.1 | *C. amalonaticus* CECT 863 | NR_104823.1 |
| *C. cronae* Tue2-1 | NZ_VOSQ00000000.1 | *C. amalonaticus* LMG 7873 | NR_118106.1 |
| *C. werkmanii* NBRC 105721 | NZ_BBMW01000009.1 | *C. youngae* GDC 1314 | NR_041527.1 |
| *C. tructae* SNU WT2 | NZ_CP038469.1 | *C. gillenii* CDC 4693-86 | NR_041697.1 |
| *C. koseri* NCTC 10786 | NZ_UAVY01000004.1 | *C. braakii* DSM 17596 | NR_117750.1 |
| *C. amalonaticus* NCTC 10805 | NZ_UFVN01000003.1 | *C. braakii* 167 *CDC* 80-57 | NR_028687.1 |
| *C. rodentium* NBRC 105723 | NZ_BBNA00000000.1 | *C. murliniae* CDC 2970-59 | MT256269.1 |
| *C. sedlakii* NBRC 105722 | NZ_BBNB00000000.1 | *C. werkmanii* CDC 0876-58 | NR_024862.1 |
| *C. youngae* NCTC 13709 | LR134485.1 | *C. cronae Tue2‑1* | NR_170426.1 |
| *C. freundii* R47 | CP040698.1 | *C. europaeus 97-79* | NR_156052.1 |
| *C. freundii* CFNIH4 | CP026231.1 | *C. freundii NBRC 12681* | NR_113596.1 |
| *C. freundii* RHBSTW-00697 | CP056336.1 | *C. freundii* organism | NR_117752.1 |
| *C. freundii* RHBSTW-00902 | CP056256.1 | *C. freundii JCM 1657* | NR_113340.1 |
| *C. freundii* RHBSTW-00935 | CP056238.1 | *C. freundii ATCC 8090* | NR_028894.1 |
| *C. freundii* RHBSTW-00942 | CP056235.1 | *C. freundii NBRC 12681* | NR_114345.1 |
| *C. freundii* R17 | CP035276.1 | ***ars*C sequences** | |
| *C. portucalensis* P10159 | CP012554.1 | *E. coli* K-12 | NZ_U00096.3 |
| *C. portucalensis* WCHCP085039 | CP043009.1 | *B. subtilis* 168 | txid224308 |
| *C. pasteurii* UMH17 | CP024676.1 | *S. bongori* 85-0051 | NZ_CP053416.1 |
| **WGS of other strains of *Enterobacteriaceae* family** | | *S. aureus* | NZ_M86824.1 |
| *E. coli* pR773 | J02591 | V. cholerae MS6 | NZ_57740780 |
| *E. coli* pR46 | U38947 | *S. marcescens* SCQ1 | NZ_CP006335.1 |
| *E. coli* chromosomes | X80057 | *P. aeruginosa* | txid1123015 |
| *K. pneumoniae* Kp52.145 | NZ_FO834906.1 | Shewanella sp. ANA-3 | txid34122 |
| *S. enterica* Newport USMARC-S3124.1 | NZ_ CP006631.1 | M. tuberculosis ATCC 25618 | NC_000962.3 |
| *Enterobacteriaceae* bacterium bta3-1 | NZ_CP004083.1 | *S. oneidensis* | WP_011072786.1 |
|  |  | *P. aeruginosa* PAO1 | NP_249641.1 |
|  |  | *N. meningitidis* | WP_002228821.1 |
|  |  | *E. coli* O157:H7 | NP_312402.1 |
|  |  | *S. enterica* | GAS71778.1 |
|  |  | *S. flexneri* 1235-66 | EIQ79235.1 |
|  |  | *S. enterica* | MBJ3556729.1 |
|  |  | *K. aerogenes* | WP_015365459.1 |
|  |  | *S. enterica* | EAA7554388.1 |
|  |  | *Klebsiella pneumoniae* | WP_117086363.1 |
|  |  | *Y. regensburgei* | WP_120815996.1 |
|  |  | *K. quasipneumoniae* | WP_180263759.1 |
|  |  | *Y. regensburgei* | WP_006818638.1 |
|  |  | *K. aerogenes* | WP_163312099.1 |
|  |  | *K. oxytoca* | WP_110272391.1 |
|  |  | *H. paralvei* | WP_095661655.1 |

**Table S5. Comparison of arsenate-reducing, Gram-negative, rod-shaped bacteria from existing literature.** MIC- minimum Inhibitory concentrations; NA – not available

| **Species** | **MIC (mM)** | | **Redox transformation** | **Isolated from** | **Type** | **Reference** |
| --- | --- | --- | --- | --- | --- | --- |
|  | **As(III)** | **As(V)** |  |  |  |  |
| *Citrobacter youngae* IITK SM2 | 14.7 | 427.1 | Reduces As(V) to As(III) | Groundwater; Kanpur, India | Facultative anaerobe | This study |
| *Citrobacter* sp. RPT | NA | 5.6 | Oxidizes As(III) to As(V) | *Pteris vittata* plants Kolli Hills, Tamil Nadu, India | Facultative anaerobe | [13] |
| *Citrobacter* sp. | NA | 266.9 | NA | Hospital; Clermont-Ferrand, France | Aerobe | [13] |
| *Citrobacter freundii* | NA | 3.87 | Reduces As(V) to As(III) | Wastewater, Sheikhupura, Pakistan | NA | [14] |
| *Citrobacter* sp. | 5 | 100 | Reduces As(V) to As(III) | Groundwater, Taiwan | Aerobe | [15] |
| *Aeromonas Salmonicida* | 16.7 | 133.5 | NA | Contaminated sediments Orbetello Lagoon, Italy | Facultative anaerobe | [16] |
| *Pseudonomas* sp. |  |  |  |  | Aerobe |  |
| *Aeromonas molluscorum* | > 16.7 |  |  |  | Facultative anaerobe |  |
| *Enterobacter* sp. | 5 | 200 | Reduces As(V) to As(III) | Groundwater, Taiwan | Facultative anaerobe | [15] |
| *Pseudomonas* sp. |  | 150 |  |  | Aerobe |  |
| *Ochrobactrum* sp. | 40 | 500 | Can reduces As(V) to As(III) | Groundwater; W.B., India | Aerobe | [17] |
| *Brevundimonas* sp. | 5 |  |  |  |  |  |
| *Pseudoxanthomas* sp. | 5 |  |  |  |  |  |
| Unclassified Rhizobiaceae | 10 | 300 |  |  |  |  |
| *Herbaspirillum* sp.GW103 | 7.34; Not reported whether As(III) or As(V) | | Reduces As(V) to As(III) | Rhizosphere soil of Phragmites austrails | Microaerobe | [18] |
| *Desulfuromonas* sp. | NA; | | Reduces As(V) to As(III) | Groundwater; W.B., India | Anaerobe | [19] |
| *Pseudoxanthomas arseniciresistens* sp. nov. | 20 | 150 | Reduces As(V) to As(III) | Groundwater; W.B., India | Aerobe to facultative anaerobe | [20] |
| *Pseudomonas* sp. M17-1 | 2 | 100 | Reduces As(V) to As(III) | Aquifer sediments; Hetao basin, China | Aerobe | [21] |
| *Pseudomonas chengduensis* | 25 | 65 | Both qualitative and quantitative; ~78% As(V) converted to As(III) | Seyed-Jalaleddin Spring Kurdistan, Iran | Aerobe | [22] |

**Table S6. Dissolved arsenic concentrations in the absence and presence of *Citrobacter youngae* IITK SM2.**

| **Conditions** | **Dissolved As added (mg L^-1^)** | **Before the reaction with strain IITK SM2** | | | **After the reaction with strain IITK SM2** | | |
| --- | --- | --- | --- | --- | --- | --- | --- |
|  |  | **As_T_ (mg L^-1^)** | **As(V) (mg L^-1^)** | **As(III) (mg L^-1^)** | **As_T_ (mg L^-1^)** | **As(V) (mg L^-1^)** | **As(III) (mg L^-1^)** |
| **As(V)-amended conditions** | 0 | bdl | bdl | bdl | bdl | bdl | bdl |
|  | 50 | 50.2 ± 2.2 | 49.5 ± 3.6 |  | 48.6 ± 6.4 | bdl | 50.3 ± 9.1 |
|  | 100 | 100.3 ± 3.5 | 103.5 ± 10.8 |  | 102.9 ± 12.1 | 0.9 ± 0.7 | 97.1 ± 5.5 |
|  | 500 | 501.6 ± 10.2 | 521 ± 23.2 |  | 494.0 ± 31.2 | 1.2 ± 0.8 | 482.6 ± 51.4 |
| **As(III)-amended conditions** | 0 | bdl | bdl | bdl | bdl | bdl | bdl |
|  | 50 | 50.2 ± 2.2 |  | 54.9 ± 7.8 | 53.3 ± 3.5 |  | 48.6 ± 1.1 |
|  | 100 | 100.3 ± 3.5 |  | 106.1 ± 2.2 | 97.2 ± 9.4 |  | 101.2 ± 2.5 |
|  | 500 | 501.6 ± 10.2 |  | 488.3 ± 36.2 | 507.2 ± 19.9 |  | 513.6 ± 61.4 |

bdl- below detection limit

**Note:**

1. Total dissolved arsenic (As_T_) was measured using ICP-MS.
2. Dissolved inorganic As(V) and As(III) were measured using IC-ICP-MS.
3. Detection limits of ICP-MS and IC-ICP-MS are listed in Table S2.

**References**

[1] D. K. Nordstrom, J. Majzlan, and E. Konigsberger, "Thermodynamic Properties for Arsenic Minerals and Aqueous Species," *Reviews in Mineralogy and Geochemistry,* vol. 79, no. 1, pp. 217-255, 2014.

[2] D. K. Nordstrom and D. G. Archer, "Arsenic thermodynamic data and environmental geochemistry," *Arsenic in Ground-water,* no. January 2003, pp. 2-25, 2003.

[3] M. M. Benjamin, *Water chemistry*. Waveland Press, 2014.

[4] B. D. Kocar *et al.*, "Integrated biogeochemical and hydrologic processes driving arsenic release from shallow sediments to groundwaters of the Mekong delta," *Appl. Geochem.,* vol. 23, no. 11, pp. 3059-3071, 2008.

[5] J. W. Bartholomew and T. Mittwer, "The gram stain," *Bacteriological Reviews,* vol. 16, no. 1, p. 1, 1952.

[6] M. Levine, "Differentiation of B. Coli and B. Aerogens on a Simplified Eosin-Methylene Blue Agar," *The Journal of Infectious Diseases,* vol. 23, no. 1, pp. 43-47, 1918.

[7] J. G. Cappuccino and C. Welsh, *Microbiology: a Laboratory Manual, Global Edition*. Pearson Higher Education & Professional Group, 2017.

[8] Y. Cong, J. Wang, Z. Chen, K. Xiong, Q. Xu, and F. Hu, "Characterization of swarming motility in *Citrobacter freundii*," *FEMS Microbiology Letters,* vol. 317, no. 2, pp. 160-171, 2011.

[9] A. J. Drummond and R. D. Waigh, "The development of microbiological methods for phytochemical screening," *Recent Research Developments in Phytochemistry,* vol. 4, pp. 143-152, 2000.

[10] S. D. Sarker, L. Nahar, and Y. Kumarasamy, "Microtitre plate-based antibacterial assay incorporating resazurin as an indicator of cell growth, and its application in the in vitro antibacterial screening of phytochemicals," *Methods,* vol. 42, no. 4, pp. 321-324, 2007/08/01/ 2007.

[11] A. A. Van Soestbergen and C. H. Lee, "Pour plates or streak plates?," *Applied Microbiology,* vol. 18, no. 6, p. 1092, 1969.

[12] M. R. Green and J. Sambrook, "Isolation and quantification of DNA," *Cold Spring Harbor Protocols,* vol. 2018, no. 6, p. pdb. top093336, 2018.

[13] T. Selvankumar *et al.*, "Isolation, identification and characterization of arsenic transforming exogenous endophytic Citrobacter sp. RPT from roots of Pteris vittata," *3 Biotech,* vol. 7, no. 4, pp. 1-6, 2017.

[14] F. R. Shakoori, I. Aziz, and A. Rehman, "Isolation and characterization of arsenic reducing bacteria from industrial effluents and their potential use in bioremediation of wastewater," *Pakistan Journal of Zoology,* vol. 42, no. 3, 2010.

[15] V. H. Liao *et al.*, "Arsenite-oxidizing and arsenate-reducing bacteria associated with arsenic-rich groundwater in Taiwan," *Journal of Contaminant Hydrology,* vol. 123, no. 1, pp. 20-29, 2011/04/01/ 2011.

[16] M. Pepi *et al.*, "Arsenic‐resistant bacteria isolated from contaminated sediments of the Orbetello Lagoon, Italy, and their characterization," *Journal of Applied Microbiology,* vol. 103, no. 6, pp. 2299-2308, 2007.

[17] A. Sarkar, S. K. Kazy, and P. Sar, "Characterization of arsenic resistant bacteria from arsenic rich groundwater of West Bengal, India," *Ecotoxicology,* vol. 22, no. 2, pp. 363-376, 2013.

[18] M. Govarthanan, S. M. Lee, S. Kamala-Kannan, and B. T. Oh, "Characterization, real-time quantification and in silico modeling of arsenate reductase (arsC) genes in arsenic-resistant *Herbaspirillum* sp. GW103," *Research in Microbiology,* vol. 166, no. 3, pp. 196-204, 2015/04/01/ 2015.

[19] T. H. Osborne, J. M. McArthur, P. K. Sikdar, and J. M. Santini, "Isolation of an Arsenate-Respiring Bacterium from a Redox Front in an Arsenic-Polluted Aquifer in West Bengal, Bengal Basin," *Environmental Science & Technology,* vol. 49, no. 7, pp. 4193-4199, 2015/04/07 2015.

[20] B. Mohapatra, P. Sar, S. K. Kazy, M. K. Maiti, and T. Satyanarayana, "Taxonomy and physiology of *Pseudoxanthomonas arseniciresistens* sp. nov., an arsenate and nitrate-reducing novel gammaproteobacterium from arsenic contaminated groundwater, India," *PloS one,* vol. 13, no. 3, p. e0193718, 2018.

[21] H. Guo, Z. Liu, S. Ding, C. Hao, W. Xiu, and W. Hou, "Arsenate reduction and mobilization in the presence of indigenous aerobic bacteria obtained from high arsenic aquifers of the Hetao basin, Inner Mongolia," *Environmental Pollution,* vol. 203, pp. 50-59, 2015/08/01/ 2015.

[22] M. A. Jebelli *et al.*, "Isolation and identification of the native population bacteria for bioremediation of high levels of arsenic from water resources," *Journal of Environmental Management,* vol. 212, pp. 39-45, 2018/04/15/ 2018.
